# Deconstructing the principles of ductal network formation in the pancreas

**DOI:** 10.1371/journal.pbio.2002842

**Published:** 2018-07-26

**Authors:** Svend Bertel Dahl-Jensen, Siham Yennek, Lydie Flasse, Hjalte List Larsen, Dror Sever, Gopal Karremore, Ivana Novak, Kim Sneppen, Anne Grapin-Botton

**Affiliations:** 1 DanStem, University of Copenhagen, Copenhagen, Denmark; 2 Niels Bohr Institute, University of Copenhagen, Copenhagen, Denmark; 3 Cell Biology and Physiology, Department of Biology, University of Copenhagen, Copenhagen, Denmark; University of Cambridge Department of Pathology, United Kingdom of Great Britain and Northern Ireland

## Abstract

The mammalian pancreas is a branched organ that does not exhibit stereotypic branching patterns, similarly to most other glands. Inside branches, it contains a network of ducts that undergo a transition from unconnected microlumen to a mesh of interconnected ducts and finally to a treelike structure. This ductal remodeling is poorly understood, both on a microscopic and macroscopic level. In this article, we quantify the network properties at different developmental stages. We find that the pancreatic network exhibits stereotypic traits at each stage and that the network properties change with time toward the most economical and optimized delivery of exocrine products into the duodenum. Using in silico modeling, we show how steps of pancreatic network development can be deconstructed into two simple rules likely to be conserved for many other glands. The early stage of the network is explained by noisy, redundant duct connection as new microlumens form. The later transition is attributed to pruning of the network based on the flux of fluid running through the pancreatic network into the duodenum.

## Author summary

In the pancreas of mammals, digestive enzymes are transported from their production site in acini (clusters of cells that secrete the enzymes) to the intestine via a network of ducts. During organ development in fetuses, the ducts initially form by the coordinated polarization of cells to form small holes, which will connect and fuse, to constitute a meshwork. This hyperconnected network further develops into a treelike structure by the time of birth. In this article, we use methods originally developed to analyze road, rail, web, or river networks to quantify the network properties at different developmental stages. We find that the pancreatic network properties are similar between individuals at specific time points but eventually change to achieve the most economical and optimized structure to deliver pancreatic juice into the duodenum. Using in silico modeling, we show how the stages of pancreatic network development follow two simple rules, which are likely to be conserved for the development of other glands. The early stage of the network is explained by noisy, redundant duct connection as new small ductal holes form. Later on, the secretion of fluid that runs through the pancreatic network into the duodenum leads to the widening of ducts with the greatest flow, while nonnecessary ducts are eliminated, akin to how river beds are formed.

## Introduction

Branching is a phenomenon that appears everywhere in life. Biological examples include tree leaves and branches [1], the arterial and venous systems [2], the liver [3], lung [4,5], kidney, and several glands such as the pancreas, the mammary [6], salivary [7], lacrimal [8], prostate [9], and meibomian glands [10]. Work carried out on independent organs suggests that several branched organs share principles, such as the importance of mesenchymal signals, and even molecules, such as a frequent use of fibroblast growth factor (FGF) sources [4,5]. However, important differences in morphogenesis also exist across organs. For example, branching is more stereotypic in the lung than in glands, in which the ductal tree differs between individuals. Though emphasis has been put on the outer shape of the branching epithelium, experiments in the pancreas [11,12] and salivary glands [13] suggest that the branching process may be driven from inside the gland when lumen form and connect into tubes.

The mature pancreas is a branched organ in which branches are formed of monolayers of cells assembled into tubes that are connected to form a treelike structure with exit into the duodenum. The pancreas is composed of three main components: acinar, ductal, and endocrine cells. Acinar cells at the terminal ends of the ductal tree secrete digestive enzymes into the ducts, which deliver them into the duodenum [14]. Ductal cells secrete water, the bicarbonate that neutralizes acidic gastric juices, and mucus that protects the ducts [15,16]. The basic pH of ductal secretions contributes to keeping the digestive enzymes inactive until they reach the duodenum. The endocrine cells reside in islets of Langerhans embedded near the ductal system and regulate glucose homeostasis by secretion into the bloodstream. In recent years, our understanding of pancreatic development has grown with increasing speed, driven by advances in both image acquisition and methods for tracking and categorizing cells.

Around embryonic day (E) 9.5 of mouse development, cells in the foregut endoderm change from a cuboidal into a columnar shape, forming the dorsal and mouse pancreatic buds [17,18]. The pancreatic progenitor cells then proliferate and form a stratified epithelium, with most cells losing their apical domain and connection to the duodenal lumen. Shortly after, clusters of cells begin forming microlumen. At E10.75, several of these microlumen exist, some of which are connected by polarized canals [12]. Isolated lumen continue to emerge and subsequently connect to this burgeoning network. This results in a plexus of interconnected ducts at E12.5 [11,12,17].

From E12.5, the now densely packed epithelium starts remodeling its ductal structure. This, combined with general expansion of the epithelium and associated ducts, results in fingerlike protrusions of plexus into the surrounding mesenchyme [12,17]. Running in parallel with this remodeling, the cells begin to segregate into domains with distinctive “tip” and “trunk” cell identities [19]. Tip domains contain cells that are progressively restricted to an acinar fate, while the trunk domain contains the endocrine/duct bipotent progenitors. Starting from E13.5, the tip cells become committed to the acinar fate and start a massive wave of proliferation, rapidly increasing the amount of acinar ends in the network [19]. At around E18.5, the network of the pancreas is more “arborized,” forming a ramified ductal network [20]. In the adult, the ducts are categorized in a rough hierarchical order of duct thickness, with the smallest intercalated ducts close to acini, intralobular ducts, and ending with the interlobular ducts separating the lobes of the pancreas [14].

We still know little about how the microlumen coalesce into a plexus. Kesavan and colleagues showed that epithelial polarity is needed for microlumen formation and maintenance of the ductal network [11]. However, a detailed description of how the microlumens form the ductal network is missing. Even less understood are the mechanisms for remodeling the ductal network from a plexus to a ramified treelike organ. Villasenor and colleagues provided invaluable insight into this process with a detailed anatomical description of the remodeling process [12]. They unveiled that the pancreas might be more stereotypic than previously believed and could identify trends in the patterns of branches. However, further understanding has been limited by a lack of quantitative measures.

In this work, we digitize the ductal network at 3 distinct phases in the development of its ductal tree. We chose E12.5 to represent the early stage with an almost completely interlinked plexus. E14.5 represents the intermediary network in which distinct cell types are appearing and plexus remodeling starts to be visible. Finally, E18.5 represents the (almost) fully mature network. We show that the pancreatic ductal system has stereotypic traits at each stage and that its remodeling can be quantified by standard network measures.

Using in silico modeling, we deconstruct the main steps of network development into a set of simple rules. We show that the creation of the early network from E10.5 to E12.5 can be explained by noisy, redundant duct formation as new microlumens form. We show that a little noise in both the amount of microlumen connections and where it connects is needed to reproduce a network similar to the E12.5. We subsequently show that the later transition from E14.5 to E18.5 can be reproduced by pruning the network based on a flux of fluid running through the pancreatic network into the duodenum. Taken together, we show that pancreatic ductal development can be conceptualized into simple, rule-based modeling that is likely of relevance to several other glands including secreting fluid.

## Results

### Digitizing the pancreatic network

**Fig 1:**
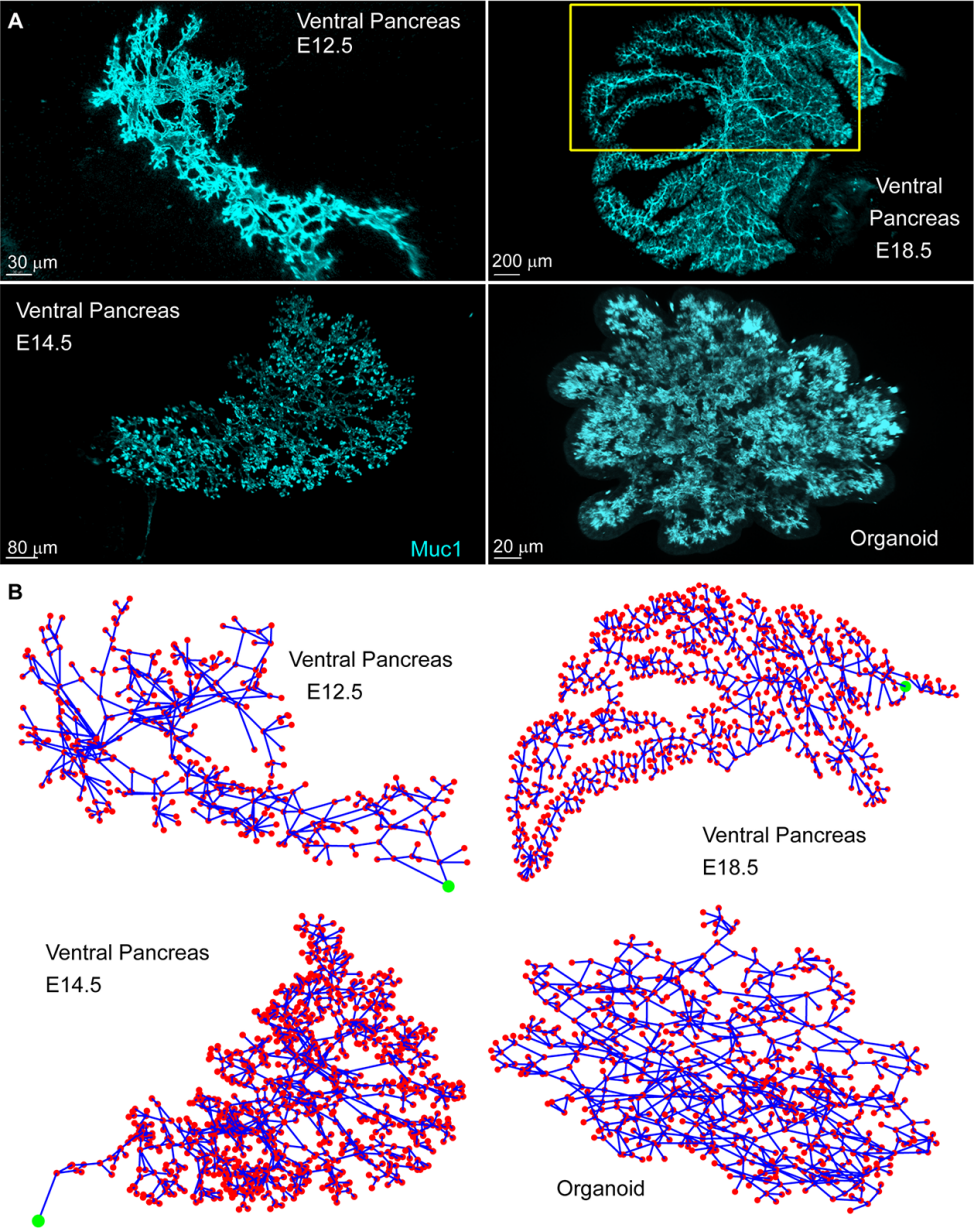
Digitized pancreas networks. Presented both in (A) their raw image format and (B) digitized. The red dots represent the mapped nodes, while the blue lines represent the mapped links. The green circle represents the exit from the pancreas (the organoids do not have an exit). The yellow box shows the mapped section of the E18.5 pancreas. Digitized data and code files “Import_Experimental_data”, “PlotNetwork” are provided in supporting information (S1 Data). E, embryonic day; Muc1, mucin1

To assess and quantify the development of the pancreatic ductal network, we first digitized it. The ducts were visualized by whole-mount immunostaining of mucin1 and e-cadherin, which respectively highlight the apical side of cells forming the ductal structure and the membranes of the cells lining the ducts. Although automatic segmentation succeeded in digitizing a large part of the network, it failed on fine ducts. Several of the ductal parameters rely on mapping all duct types. The networks were therefore manually skeletonized at different time points by mapping the terminal ends and intersections of the ducts. Thereby, we defined nodes and linked them by edges following the ductal system (S1 Fig). The method is labor intensive, and we therefore largely focused on the ventral pancreas, as it is almost entirely planar at its later developmental stages and is therefore easier to map manually. Some comparisons with the dorsal pancreas were done at E14.5 to test whether the network was different. The resulting networks (Fig 1) closely resemble the pancreatic structure reported in previous studies [11,12,17,20]. The network appears as a plexus at E12.5 and E14.5 and ends as a more treelike structure at E18.5.

### Quantifying network properties and their temporal evolution

**Fig 2:**
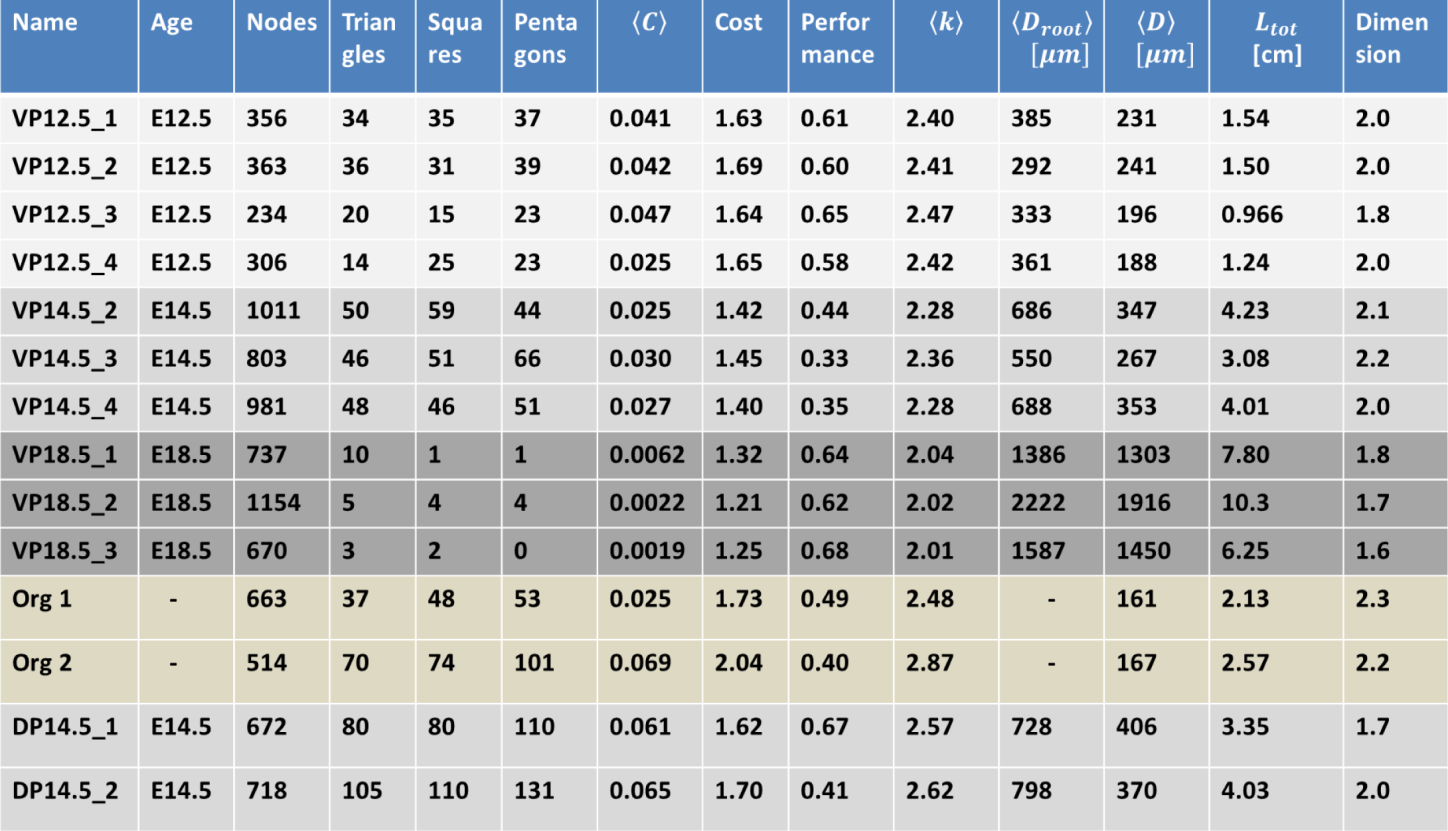
Network properties for the in vivo samples. < …> denotes the average of a value for every node in the network. C is the clustering coefficient for a given node. k is node degree. D_root_ is a node's distance to the root node through the network. D is the average distance of a node to every other node. L_tot_ is the total amount of link length in the entire network. Digitized data and code files “Import_Experimental_data”, “ConvertToAdjMat”, “ConvertToAdjList”, “NetworkProp”, “NetworkShapes”, “FindTriangles”, “Remove_kinks” are provided in supporting information (S1 Data). DP, dorsal pancreas; VP, ventral pancreas.

The digitization of the pancreatic duct system enables us to derive its network properties based on nodes and links that form the network (Fig 2). A node’s degree k is the amount of connections it has to other nodes. The polygonal features formed by the nodes and their links are derived by counting network limit cycles and give a measure of how interlinked the network is [21]. The average clustering coefficient 〈*C*〉 (<…> denotes average) relates to the number of triangles found in the network [22]. In addition, we consider the cost of the network in terms of redundancy of paths from one point to another. This was quantified by comparing the network in question to its (euclidian) minimum spanning tree (MST) obtained by connecting the nodes in the spatial location with links that minimize the total length of all links in the network [23,24]. A similar comparison quantified network performance, defined by the transport distance along the network between all pairs of nodes, normalized by the MST.

**Fig 3:**
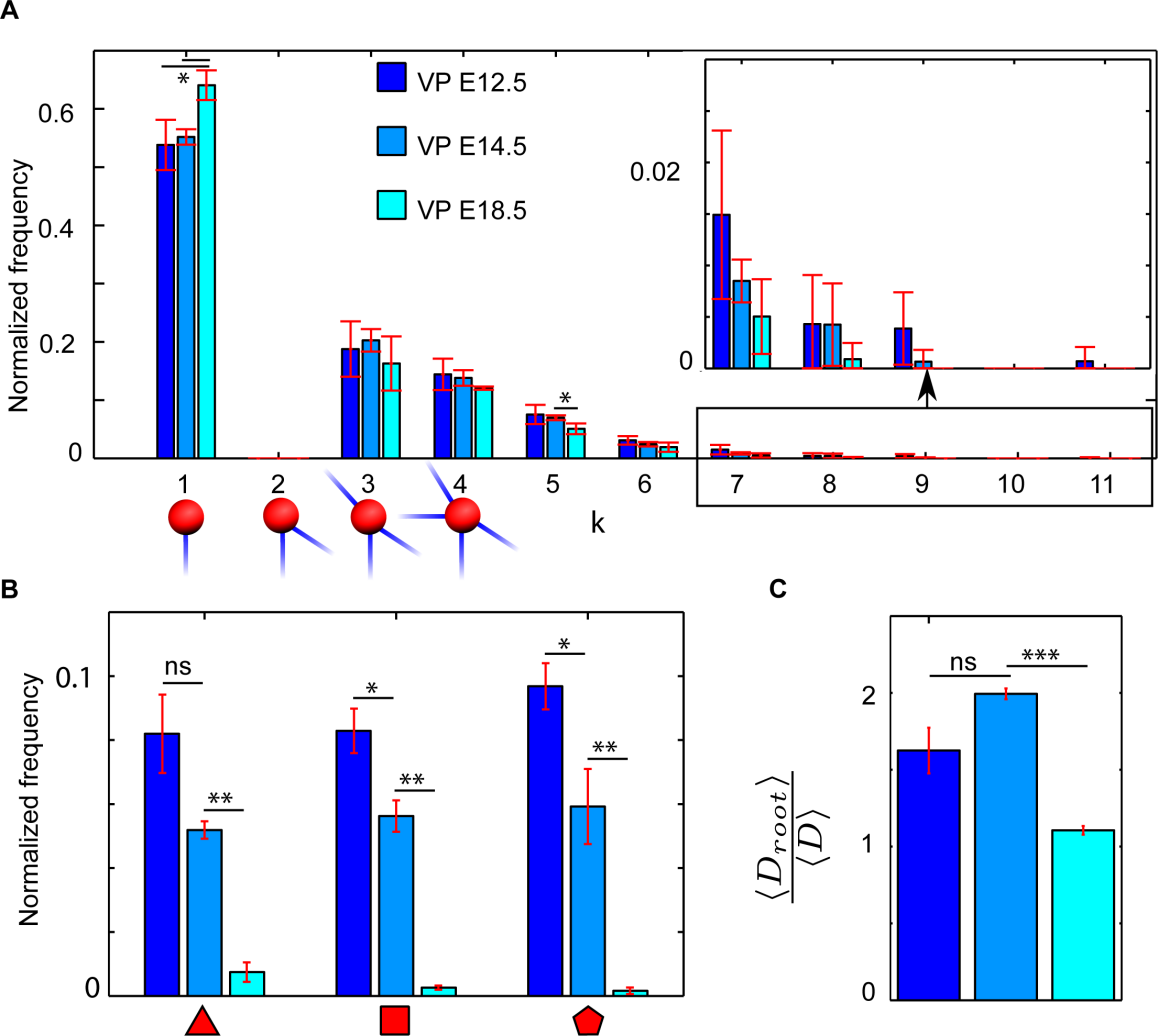
Time evolution of pancreas network features. (A) Distribution of network degree for the different pancreas stages. A node of degree 1 corresponds to a terminal end. (B) Distribution of polygonal features for the different pancreas stages. (C) Average distance of all nodes to the exit scaled by the average distance between all nodes for the different pancreas stages. The absence of nodes with degree 2 is a result of the technique used to digitize the pancreas network, as those nodes merely represent a continuation of ducts. Error bars represent SEM. Digitized data and code files “Import_Experimental_data”, “ConvertToAdjMat”, “ConvertToAdjList”, “NetworkProp”, “NetworkShapes”, “FindTriangles”,”Remove_kinks” are provided in supporting information (S1 Data). E, embryonic day; ns, not significant; VP, ventral pancreas.

The E12.5 and E14.5 pancreas networks contain many polygonal features (short loops), have a cost above 1, and have a performance below 1, all features of an overconnected network [24]. At these stages, there is more than one path from a point to the duodenal exit. Both the E12.5 and E14.5 networks have a dimension close to 2 (Fig 2, S2 Fig), highlighting that the planarity that is visible at E18.5 is also present in the network from early developmental stages. The dorsal pancreas, mapped at E14.5, is also an overconnected network. The dorsal pancreas has more redundant ducts than the ventral pancreas at this stage, evident from its number of polygons, average degree, and clustering coefficient. The dimension of the network remains close to 2, suggesting that the dorsal pancreas is also planar. The ventral E14.5 network has a slightly lower cost, worse performance, and slightly fewer polygons than the E12.5 after normalization to network size, which suggests that some redundant ducts may start to be eliminated. The E18.5 network has almost no polygons and a cost of 1.2. The ventral network was mapped on about half of the ventral pancreas at E18.5, always on the same area. A single E18.5 ventral pancreas has been fully mapped to see the shift in network properties from the partially mapped E18.5 (S3 Fig). Nearly all features analyzed were similar, with the exception of performance. This finding lends credibility to the use of partially mapped E18.5 ventral pancreata, with the caveat that the true performance of these networks might be even better, closer to 0.4, based on the whole ventral part analysis. This mature network contains almost no redundant connections. The network undergoes drastic changes as the pancreas matures (E12.5 → E18.5), with a loss of almost every polygonal structure and an order of magnitude shift in clustering coefficient (Figs 2 and 3).

The size of the network increases as seen in the total length *L*_*tot*_ of the ductal system (bearing in mind that only half of the E18.5 networks were mapped). The average distance to the network exit 〈*D*_*root*_〉 increases with development, meaning that the network expands away from the exit node. The average distances between all nodes 〈*D*〉 increases even more than to the common exit node, reflecting that distances in the early development are reduced by redundant connections. The system loses its redundant connections and thereby has a decrease in cost and a shift in network dimension from 2 to 1.7. This transformation agrees with the visual evidence that the pancreas prunes its structure to a treelike structure [12]. The changes from E12.5 to E14.5 are less pronounced, although there is a significant loss of local loops (polygons) and a slight decrease in average degree 〈*k*〉. However, the absolute number of polygonal features of the E14.5 network is higher than at E12.5. This suggests that either pruning of links takes place alongside growth at the E14.5 stage or that the newly formed network forms without loops. The existence of loops at the network periphery suggests that the newly grown network also forms with redundant ducts while pruning has been initiated (S4 Fig). None of the digitized networks resembled their randomized counterparts, suggesting that there are rules behind their formation (S5 Fig).

We visualize some of these network transformations in (Fig 3). As the pancreas matures to E18.5, the network gains more nodes with degree 1 (terminal end) compared to earlier stages. Even at the mature stage, the network retains a few nodes with degree 5 and higher. Fig 3B also visualizes the normalized distribution of polygonal features in the network as the pancreas matures. Each developmental stage has a defining amount of such loops. It is therefore possible to separate each pancreatic stage based on its polygons. Another and more global network characterization is the average distance to the exit normalized by the average distance between every node 〈*D*_*root*_〉/〈*D*〉 (Fig 3C). This property decreases substantially as the pancreas matures from stage E14.5 to E18.5. Strikingly, the average distance to the exit of the pancreas approaches the average distance between nodes. This again demonstrates the development toward a branched treelike structure, with the exit node as the common center. Taken together, our analysis shows that although the branches of the pancreas are different between individual mice [12], the pancreatic network structure is stereotypic at each stage and changes systematically as the pancreas matures.

### Network creation by redundant linking

**Fig 4:**
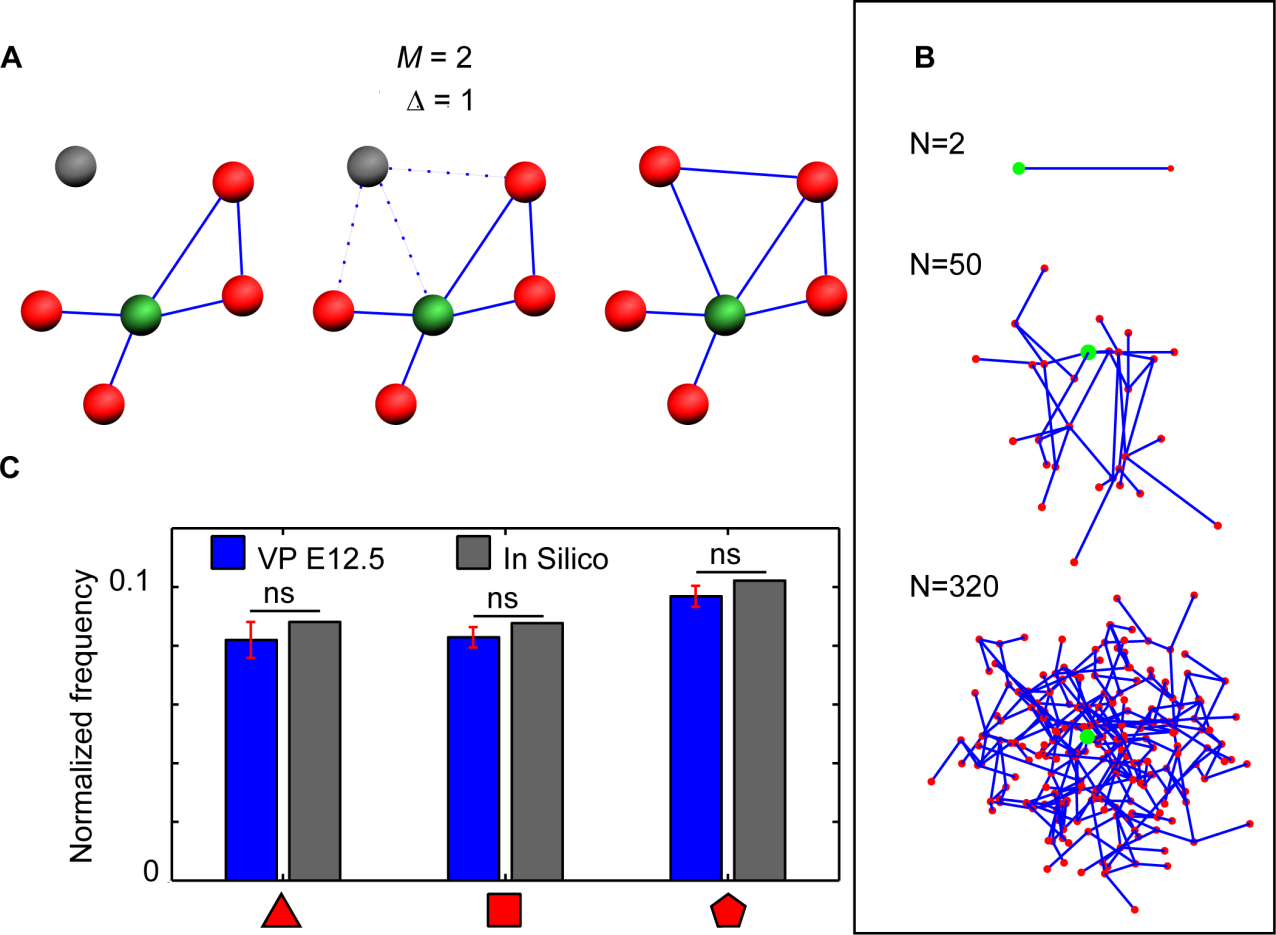
In silico network creation of ventral E12.5 networks. (A) Schematic of the in silico model. Step I: A node is created in the neighborhood of the existing network. Step II: The created node links to M nodes drawn from a pool of the nearest M + Δ nodes. Step III: The model reiterates until the desired node amount has been reached. (B) Network evolution of the in silico model from 2 nodes to 320 nodes. (C) Distribution of polygonal features for the in silico network nodes and the E12.5 pancreas network. Error bars represent SEM. Code files “NetworkCreation”, “ConvertToAdjMat”, “ConvertToAdjList”, “PlotNetwork”, “NetworkShapes”, “NetworkProp”, “FindTriangles”,”Remove_kinks” are provided in supporting information (S1 Data). E, embryonic day; ns, not significant; VP, ventral pancreas.

In order to deconstruct the formation and maturation of the ductal network, we condensed our observations into a few principles and implemented these in silico (Fig 4). First, we describe the formation of the network in terms of a growth model, starting with a single lumen node (LN). LNs are subsequently added at a random position within *r*_*CM*_ + 0.5 of the existing LNs’ center of mass, where *r*_*CM*_ is the distance from the LNs’ center of mass to the most distant point in the network. These newly added nodes are then connected to the already generated network. Each new node forms M + 1 links with the existing network, where M is drawn from a Poisson distribution with mean λ. The assigned links are then attached to the neighboring LNs drawn from a pool of the M + 1 + Δ closest LNs, where Δ is parametrizing the spatial specificity for local links formation. Fig 4C shows the best fit together with the in vivo data for the E12.5 networks. In the supporting information, we show that an expanded parameter analysis of the in silico network approximately recapitulates the observed properties of the E12.5 pancreas network, provided that Δ is about 1. The cost measures are the limiting factor for larger Δ values (S6 Fig). Further, it seems that λ = 0.25 yields the best fit, which means that simulations best fit the biological data when new nodes only connect to slightly more than 1 node. A higher λ results in too many triangles and a too high cost measure of the network (S6 and S7 Figs), whereas a lower λ results in too few loops of any length. Therefore, in order to achieve the observed network, some noise is needed in the number of formed connections between new nodes and where they connect. We notice that the best fit does not completely pass every statistical test for all measures. These discrepancies might be explained by the lack of a growth direction in the model, which results in a roughly spherical structure of the simulated 3D network. Some secretory organs, such as the kidney, exhibit an initial phase of stereotyped duct formation followed by less stereotyped processes [25,26]. Though this has not been observed in the early pancreas [12], this may have been overlooked. We thus attempted to initialize the network from small initial binary/dichotomic patterns (L-system) of 4, 20, and 100 out of the nodes eventually generated. The decrease in polygons becomes significant only when the initial L-system makes up a large fraction of the whole network (100 out of about 320) (S8 Fig).

### Flux-based pruning of the network

**Fig 5:**
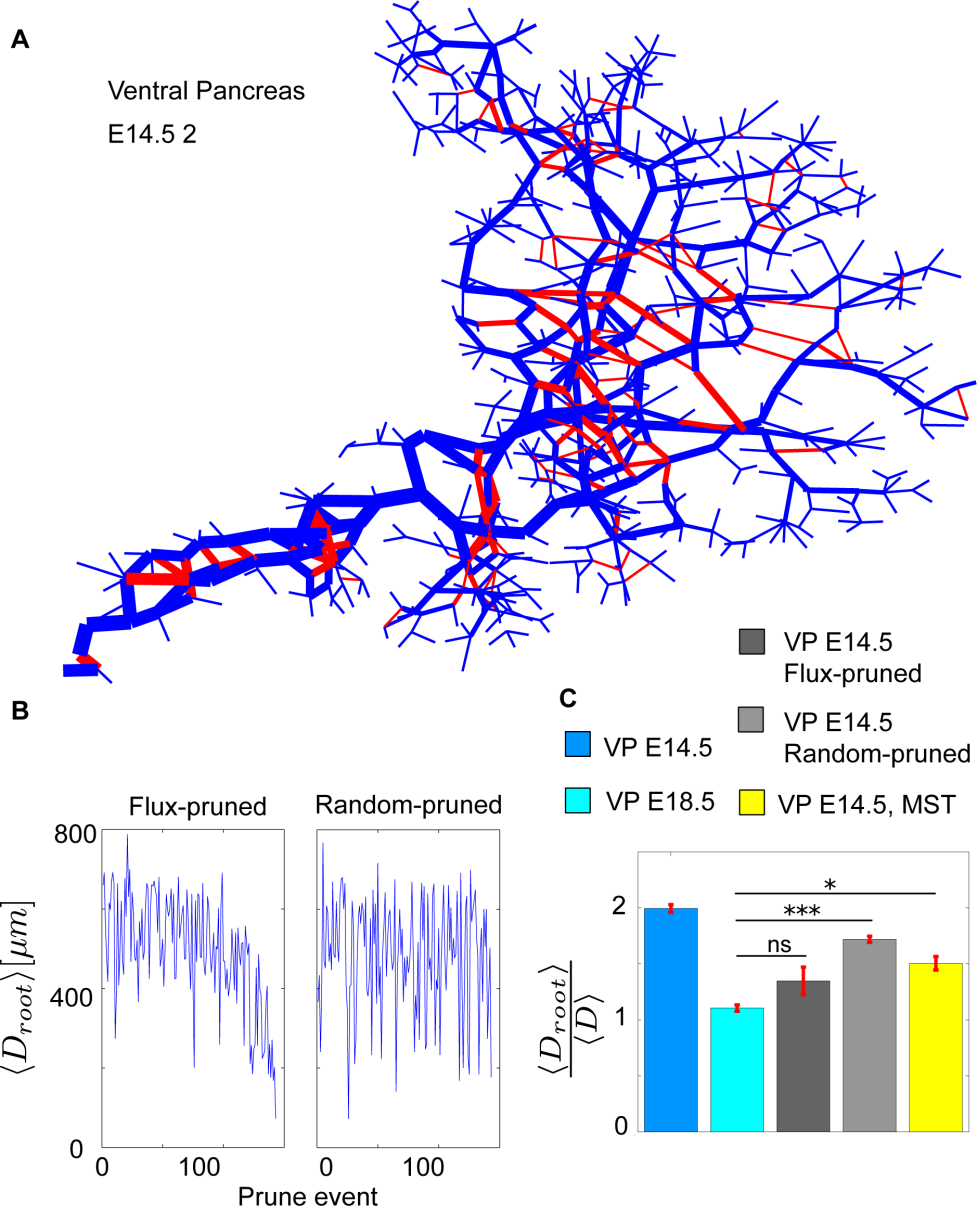
Flux-based pruning of the VP14.5 networks. (A) The logarithm of the normalized flux at steady state of the E14.5 2 pancreas network. Thicker links indicate a higher flux. The highest flux is closest to the exit, with some interlinking nodes having very low flux. The links highlighted red are pruned by the pruning mechanism of least flux. (B) The pruning event's distance from the exit as pruning progresses for flux-based pruning and random pruning. (C) Average distance of all nodes to the exit scaled by the average distance between all nodes shown for the networks of E14.5, E18.5, the in silico pruned E14.5, and the E14.5 MST. Error bars represent SEM. Code files “Import_Experimental_data”, “DiffusionOnNetwork”, “PruneBasedOnFlux”, “SnapShot”, “ConvertToAdjMat”, “ConvertToAdjList”, “NetworkProp”, “NetworkShapes”, “FindTriangles”, “Remove_kinks” are provided in supporting information (S1 Data). E, embryonic day; MST, minimum spanning tree; ns, not significant; VP, ventral pancreas.

The ductal network of the E12.5 pancreas has an inhomogeneous duct thickness that does not indicate a hierarchy between the main duct and its terminal ends [12]. In contrast, the ductal network at E14.5 exhibits some hierarchy of duct diameter, with wider ducts close to the exit, making it possible to identify a difference between terminal ends and the main duct [12]. This hierarchy is even more distinct at the E18.5 stage, in which the main duct and the acini ends are easily identified [12]. This suggests that duct diameter relates to fluid running through the developing pancreas. We hypothesized that fluid running through the network may contribute to other time evolutions of the ductal network such as the pruning of redundant ducts. We postulated that, as the pancreas matures, redundant ducts would compete, and the smallest of competing ducts may be remodeled or merged. In order to assess this flux-based mechanism for pruning of the network, we constructed a simple in silico model (S9A Fig). The model uses the already digitized E14.5 pancreatic networks as its input. Fluid is added at the terminal ends of the network continually and is drained at the exit to the duodenum. This assumes that the acinar cells are the main contributors of fluid in the developing pancreas, but this assumption can be relaxed by simulating ductal secretion on every node, without significant change of our results (see Fig 5, S10 Fig). There is in fact evidence that ductal cells also secrete fluid during development [27]. The network is then allowed to reach steady state. This simulation provides a measure of the flux through all the ductal links (Figs 5A and S10A). We see that the high flux links lie in the inner pancreas structure, and the highest fluxes are found close to the exit of the pancreas (S11 Fig). This would impose a higher hydrostatic pressure and flow, which promotes widening of the ducts, in agreement with the observation that the largest ducts of the pancreas are closest to converging points and to the exit [12]. Similar phenomena have been reported during the transition from networks to trees in blood vessels [28–31]. According to Poiseuille’s law, a high flux of fluid is expected to result in a high internal pressure, which is lessened by increasing duct width, equilibrating at steady state to the same pressure for every duct (see Materials and methods). In agreement with this hypothesis, we show that the drained basin (approximated to the total number of total nodes or duct length upstream of a given duct) is proportional to the cube of duct diameter (S11 Fig). This is exactly what is to be expected from laminar flow in a duct system with draining (see Materials and methods).

Our assumption is further that ducts that carry low or no flow tend to be eliminated. The second part of the model therefore consists of pruning the network, removing redundant links with the lowest flux. A link is considered redundant if its removal does not fragment the network (S9A Fig). This process is repeated, removing subsequent redundant links until the network is a perfect treelike structure (Fig 5). We observe that flux-based pruning results in the network being pruned from its periphery and progressively toward the center/outlet (Fig 5B, S10B Fig). This result agrees with the experimentally observed network structure [32].

We further compare the flux-pruned network with a network that instead is pruned by randomly removing redundant links regardless of their flux (Fig 5, S10B Fig). Fig 5C quantifies that the flux-pruned network matches the mature pancreas significantly better than the random-pruned network. The random-pruned network does not correctly prioritize the exit to the duodenum (Fig 5C), and as a result, it will be less efficient at transporting fluid to the exit.

### Experimentally induced lack of exit prevents pruning

We previously established an in vitro 3-dimensional (3D) culture model in which small clusters of pancreatic progenitors proliferate, differentiate, and self-organize to form a pancreas-like structure [33]. The forming organoids branch and form ducts after 7 days of culture [33,34]. By whole-mount immunocytochemistry detection of the apical marker mucin, we observe that these organoids form a network of pancreatic ducts. This network is isotropic and lacks an exit point. Although the organoids are extracted from E10.5 pancreas and kept for 7 days in culture, they do not recapitulate the features of a network that resemble the one seen in the pancreas at E18.5. They do establish lumen and connect them into a network, but the network in the organoid exhibits a large number of loops, high cost, low performance, and high average connectivity (Figs 1 and 2). Its high cost and low performance suggest a level of connectivity that is even higher than the E12.5 pancreas. A network devoid of exit point is not exposed to a net flux, even if it is actively secreting, which is suggested by the observation that with time, the lumens widen to eventually form cysts at day 10 of culture [33]. Taken together, this suggests that in the absence of flux, pruning does not occur.

### Secretion of fluid during embryogenesis

**Fig 6:**
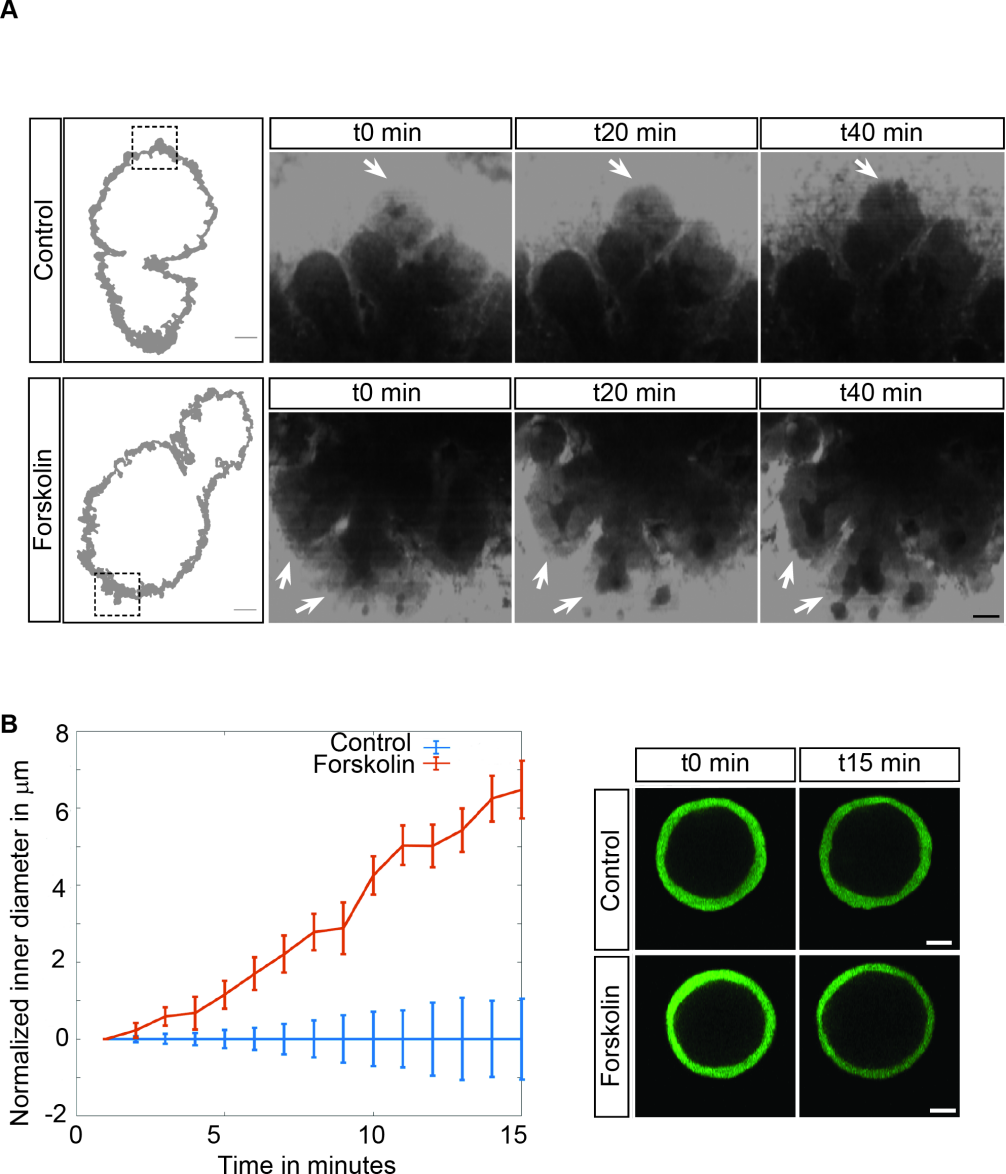
Swelling assays reveal forskolin-induced secretion. (A) E12.5 pancreata cultured in vitro for 1 day and submitted to forskolin treatment activating the CFTR channels undergo lumen swelling. The lumen appears as a dark shadow, and some are highlighted with arrows (B). Ductal spheres generated from E12.5 pancreata swell upon treatment with forskolin, as compared to untreated controls. CFTR, cystic fibrosis transmembrane conductance regulator; E, embryonic day.

Pancreatic secretion and flux have not been studied in the embryo. The fluid secreted by the pancreas in the adult originates from ductal cells, for the most part, and from acinar cells [16,35]. Many of the channels and transporters involved in fluid secretion have been uncovered. Using transcriptional profiling, we found that many were already expressed at E10.5, E12.5, and 14.5 (S12 Fig). Several important players were up-regulated between E10.5 and E12.5 at the time of microlumen formation—notably, the apical chloride channel cystic fibrosis transmembrane conductance regulator (CFTR), which is coupled to the anion exchanger solute carrier family 26 member 6 (Slc26a6) for ductal bicarbonate secretion, as well as the calcium-activated chloride channel anoctamin-1 (Ano1)/transmembrane member 16a (Tmem16a), active also in adult acinar and ductal cells [16,35,36]. These are the main channels secreting ions and osmotically driving the water flow in the adult pancreas and in the salivary gland. The main pancreatic water channel, aquaporin 1, is also strongly up-regulated between E10.5 and E12.5, as well as the secretin receptor, which activates ductal secretion in adult. To test whether the secretory machinery is active, we stimulated CFTR channels using forskolin on E12.5 pancreatic explants (Fig 6A). This led to a dilation of ductal/acinar lumen. We also observed a dilation on spheres produced from E12.5 progenitors (Fig 6B). These spheres are mostly formed of ductal cells. These experiments reveal that the pancreatic cells secrete fluid as early as E12.5 and clarify the molecular machinery used.

## Discussion

Here, we show that mapping the pancreatic network unveils previously unknown features of pancreatic development. This work on a specific organ is likely to be relevant to other glands that also exhibit connecting microlumen, such as the salivary gland and more generally glands that secrete fluid. The organ-forming rules we propose here are different from the more deterministic lung branching program or the random walk proposed in the mammary gland [5,37]. We reveal that although the pancreatic branches are not stereotypic at different orders in the network and between individual mice, the pancreatic network has stereotypic properties at different developmental stages between E12.5 and E18.5. We show that the transition of the pancreatic ductal network from an interlinked mesh to a treelike structure is reflected in changing network properties. Furthermore, we uncover that this transition optimizes the distance from the acinar ends to the duodenum while reducing redundancy in the ductal network. The development of the pancreatic tubular network can be described by redundant lumen connection followed by flux-based pruning. Redundant lumen connection is simulated with a simple growth model in which new lumen appear close to the established pancreas network and immediately connect to the nearest lumen of the network. The model recapitulates the network traits of the E12.5 pancreas, but the molecular mechanisms that drive network connection—that is, the alignment of cells with coordinated apicobasal polarity in continuous tubes—are currently unknown. The network creation model predicts that if new microlumen connect to every node (λ = ∞) when they appear, then the network should form a single large lumen. This is possibly what happens in *Pdx1 −/−* mouse strains [38]. In this strain, the network has a single lumen at E11.5. At later developmental stages, microlumen do appear and connect to this monolumen, expanding it to a single large lumen [38].

Even a small reduction of the average connectivity λ has a profound effect on the in silico network, in particular a loss of polygonal features, a decrease in cost (fewer redundant links), and lower performance (longer distance between nodes). A reduction in λ may be the phenotype that was observed in the *p120 −/−* mice [39]. These mice have fewer ducts and larger ducts than their wild-type counterparts. In addition, the p120 −/− pancreas also contains fewer branches, suggesting a correlation between the inner ductal network and the outer branching morphology in the pancreas [39].

The subsequent model for flux-based pruning is a zero-parameter model that assumes that pruning of the ducts is solely based on the minimal assumption that redundant links with low flux are removed first. Thereby, we recapitulate the transition of an E14.5 pancreas network into a treelike network resembling the more mature E18.5 structure. Villasenor and colleagues qualitatively characterized the developing morphology of the pancreatic ductal network and its outer morphology [12]. Our work expands this description by providing quantitative measures of the developing ductal network. With our dataset, it is possible to compare the pancreatic network to other transportation networks such as, for example, rivers [40–43], infrastructure [24], and blood vessels [44]. This comparison suggests some similarities to other networks transporting fluids. As seen in these networks, we observe wider ducts close to the network exit. Our quantitative measurements of duct diameter reveal that the drained basin, which is expected to be proportional to flow, is proportional to the cube of duct diameter in a manner similar to blood vessels.

Though we show that network restructuring is best modeled by the elimination of ducts with the lowest flow when alternative paths exist, it raises questions as to how the ducts are eliminated. Cell death is unlikely, as there is relatively little cell death during pancreas development, and they do not form patterns of aligned cells. It is more likely that cells are rearranged and recycled to widen or elongate contiguous ducts. The recent observation that Afadin and Ras homolog gene family member A (RhoA), acting upstream of microfilament dynamics, are needed for loop elimination is in agreement with this hypothesis [45]. This raises questions as to how cells sense the lowest flow and the fact that the duct is supernumerary. It is unlikely that the cells measure an absolute amount of flow, as ducts with different flows are pruned in the model. A likely scenario is that the ducts with high flow build fluid pressure, leading to a response that widens these ducts, and that this promotes pulling forces and cell redistribution from adjacent ducts. For nonsupernumerary ducts, the initiation of closure would be expected to lead to pressure increase and reopening [46]. A flow increase may be sensed by multiple systems, including multiple ion channels, adhesion and cytoskeletal proteins, cilia, caveolae, and the membrane bilayer itself [47]. While this has been studied mainly in the blood vessels and largely in the adult, similar systems may be used in the pancreas. The elements of the flow that are sensed may be shear stress, circumferential stretch, or, though less likely, variations in the flow.

Among these sensors, only primary cilia have been shown to affect the pancreatic ductal network. Ductal cells have primary cilia on their apical side, while acinar cells do not [48,49]. Bending of cilium caused by fluid movement has been shown to cause an activation of the calcium channels in kidney tubules, affecting a number of downstream targets [50–52]. Disruption of cilia affects the structure of ducts in both the kidney and the pancreas; local ductal enlargements have been reported, but the ductal network has not been studied in whole mount, and we therefore do not know whether the resolution from a plexus to a tree is affected.

Specific pancreatic inactivation of kinesin family member 3a (Kif3a), a molecular motor used to build the cilia during development, led to cyst formation and duct enlargement. However, when cilia formation was inactivated 4 weeks after birth, no phenotype was observed [53]. This suggests that, like in the kidney, primary cilia play an important role in developing the pancreatic duct network but have a lesser role in maintaining it. Furthermore, when Kif3a inactivation was limited to the acinar and endocrine cells, no phenotype was observed [53]. Therefore, only the primary cilia of the ductal cells play an important role in pancreas tubulogenesis during development. In a similar manner, inactivation of Tg737/Polaris [54], a protein that promotes cilia assembly, leads to cyst formation. Several other mutants affect cilia and lead to cystic ducts, including hepatocyte nuclear factor 6 (HNF-6) −/− mice [55], specific pancreatic inactivation of hepatocyte nuclear factor 1 beta (HNF1B) [27], as well as bicaudal C homolog 1 (BICC1) inactivation mutants [56]. In all these mutants, the defects reported are ductal enlargements, and it would be important to know whether other ductal parameters studied here are affected.

The model also predicts that fluid must run though the pancreas from E14.5 or even earlier in order to prune correctly. Secretion in the adult pancreas originates from ductal and acinar cells. We show that the main drivers of ductal secretion—TMEM16, CFTR, and aquaporin 1—are already expressed by E12.5, and duct enlargement upon forskolin exposure suggests ductal secretion. Since spheres are primarily made of bipotent progenitors [33], we surmise that they produce a large part of the fluid. At E12.5, there are no acinar cells in the pancreas yet, but acinar cells may contribute after they differentiate. CFTR is expressed at relatively low levels in the ducts at these stages, and its importance in secretion in mice is less important than in human. It has been proposed that this is due to functional overlap with calcium-activated chloride channels (TMEM16A/ANO1) in mice [57,58]. In human and pigs, CFTR mutations are already symptomatic in the fetal pancreas as early as 19 weeks of pregnancy. Ductal obstruction and enlargement have been reported antenatally in these models, providing a natural circumstance [59] [60] suggesting that secretion starts before birth as well [61]. We did not observe dilation in explant nor spheres with secretin, cholecystokinin (CCK), or carbachol, which respectively activate secretion in adult ducts, acini, and both, even though their receptors are expressed during pregnancy. [17,56]. An interesting mutant lends support to the hypothesis that secretion test runs lead to network remodeling. Indeed, the pancreas-specific inactivation of the transcription factor nuclear receptor subfamily 5 group A member 2 (NR5A2) prevents the formation of acinar cells and intercalated ducts [62]. In this mutant, the ductal network remains hyperconnected at birth, suggesting that acinar or terminal ducts control network pruning, possibly via their secretion.

Our study suggests more elaborate tests of our flux pruning scenario. The first is to obstruct the pancreas exit into the duodenum during normal development, a difficult experiment in vivo in embryos and for which no spontaneous incidence has been reported. In the same spirit, organoids, which do not have an outlet, do not exhibit network optimization. We experimentally tried to use capillaries to create an artificial outlet, but inserting them in ducts and preventing clogging prevented success. The organoid experiment shows that the formation of the ductal meshwork occurs independently of the formation of a blood vessel network, though eventually, blood vessels are enriched along the pancreatic epithelial branches of the tree, and cross-talk is known to exist between the two tissues [63–65].

Our work provides new methods and a new framework to evaluate the formation of ductal networks in organs and sets the stage to evaluate structural defects in mutants to decipher the cellular events underlying network remodeling. Moreover, it paves the way to analyzing similarities and differences in the secretory network of other glands.

## Materials and methods

### Ethics statement

The Danish Veterinary Office approved the animal experiments performed under license number 2014-15-2934-01008.

### Explant, organoid, and sphere culture

Timed-pregnant ICR mice were killed at E10.5 (for organoids and spheres) or E12.5 (for spheres), and the cells of embryonic pancreases were recovered, dispersed, and seeded in Matrigel as described previously [33,34,66]. Organoids were cultured for 7 days and spheres for 4 days prior to imaging. For explants, E12.5 pancreases were cultured overnight on fibronectin-coated glass-bottom plates, as described previously, prior to live image acquisition [67].

### Immunohistochemistry

Whole-mount immunostaining was performed for pancreata harvested at E12.5, E14.5 (dorsal pancreata only), and E18.5 and organoids harvested at day 7 in vitro as previously described [26]. Samples were fixed with 4% paraformaldehyde at 4°C and then washed in phosphate-buffered saline (PBS). Samples were dehydrated stepwise in 33%, 66%, and 100% methanol for 15 minutes at each step and stored at −20°C until later use. For autofluorescence quenching, they were incubated with freshly prepared Methanol:DMSO:H2O2 (2:1:3, 15% H2O2) at room temperature (RT) for 12–24 hours. Samples were washed twice in 100% methanol for 30 minutes at RT and brought to –80°C 3–5 times for at least 1 hour each time and back to RT to make antigens in the deeper parts accessible. Samples were rehydrated stepwise in Methanol/TBST (50 mM Tris-Hcl pH 7.4, 150 mM NaCl, 0.1% TritonX-100) 33%, 66%, and 100% for 15 minutes at each step at RT. After blocking overnight at 4°C in CASBLOC blocking solution, samples were incubated with primary antibodies (Ecadherin [1/200] # 610181, BD transduction laboratories and Mucin1 [1/200] #HM-1630-P0, Thermo Fisher) diluted in CAS-Block Histochemical Reagent, # 8120, Thermo Fisher for 24–48 hours. Then, samples were washed overnight in TBST and incubated with secondary (goat anti-A. hamster biotinylated [1/1,000], Invitrogen) and tertiary antibodies (donkey anti-mouse Alexa 488 [1/200] and strepatividin Alexa 647 [1/800] Invitrogen) and for 24–48 hours followed by overnight washing in TBST. Stained samples were dehydrated stepwise in Methanol as previously described and cleared in a solution of 1:2 Benzyle Alcohol and Benzyle Benzoate (BABB) for 12–24 hours prior to imaging.

### Image acquisition and reconstruction

Cleared samples were subsequently mounted in glass concavity slides and submerged completely in BABB to maintain refractive index matching and sample transparency. Cleared samples were imaged using a Leica SP8 confocal microscope with a 20X/0.75 oil immersion (most samples) or a 10× air (E18.5) objective at 1024 × 1024 resolution. Samples were imaged in an 8-bit format unless otherwise indicated. Images were stitched and reconstructed into 3D images using the Imaris software (Bitplane).

For live imaging of explants and spheres, we used a Zeiss LSM780 confocal microscope with a 10× air objective. Calcein 10 μM was used as background stain and incubated for 30–60 minutes and rinsed before imaging. Independent samples were imaged at least in triplicate for 40 minutes, 1 image every 2 minutes, after addition of the drug tested. We tested 10 μM Forskolin, 1 μM Ionomycin, 10 nM secretin + 10 μM Carbachol.

### Image analysis and quantification

The inner diameter was automatically segmented and measured for each sphere at each time point using Lumen Thickness Detector script (https://github.com/gopalrk/Lumen_Detector_and_Quantification). The value of the inner diameter at t was first normalized to t0 and then normalized to control over time (*n* = 2 independent experiments; the total number of analyzed spheres is 68 for forskolin treatment and 59 for control). Statistically different diameter between t0 and t15 in forskolin condition and between t15 forskolin versus control; Mann–Whitney U test, *p* < 0.001.

### Statistical methods

Every mean and standard error of mean (SEM) shown in the paper is based on the data shown in Fig 2. All statistical tests in the paper are 2-sample, 2-tailed *t* tests and thus assume the data have a Gaussian distribution, which we cannot test. The annotation for *p*-values are as follows: *p < 0.05, **p < 0.01, ***p < 0.0001.

### Transcriptome analysis

Pancreata were isolated at E10.5, E12.5, or E14.5 and lysed with lysis buffer RLT, and RNA was purified following the manufacturer instructions (RNeasy Plus Micro Kit, #74034, Qiagen). RNA quality was assessed using an Agilent 2100 Bioanalyzer, following the manufacturer instructions (Agilent RNA 6000 Pico Kit # 5067–1513). Extracted RNA (700 pg) was amplified using Ovation Pico SL WTA system V2 (# 3312–48, Nugen). Samples were labeled with SureTag DNA labeling kit (#5190–3391, Agilent Technologies), run on SurePrint G3 Mouse Gene Exp v2 Array (# G4852B, Agilent Technologies), and read by a SureScan Microarray Scanner (Agilent Technologies).

### Calculation of network properties

In the section below, we describe the calculus needed to derive the network properties shown in Fig 2, S3 Fig, and S5 Fig.

A network of N nodes and E links (edges) can be represented by its N × N adjacency matrix A

$$A(i,j)=\left\{ \begin{array}{l} 1,if\;node\;i\;is\;connected\;to\;node\;j\\ \;0,\;otherwise \end{array}.\right.$$

For an undirected network, A(i,j) = A(j,i). Nodes in the networks do not connect to themselves, *A(i*,*i) = 0*, and so the trace of A is always zero $\sum_{i=1}^{N}A(i,i)=0$.

A modified adjacency matrix can be constructed, called the weighted adjacency matrix *A*_*w*_:

$$A_{w}(i,j)=A(i,j)d(i,j),$$

where d is the euclidian distance between node i and node j. The total length of the network *l*_*T*_ is defined as the sum of all weighted links:

$$L_{tot}=\frac{1}{2}\sum_{i=1}^{N}\sum_{j=1}^{N}A_{w}(i,j).$$

The cost of the network is the total length of all the links in the network compared to the same value of the euclidian MST composed of the same nodes [24].

$$C=\frac{L_{tot}}{L_{tot}^{MST}}$$

The performance of the network is the average shortest path between every node compare to the same value of the MST:

$$P=\frac{\langle l\rangle }{\langle l^{MST}\rangle },$$

where *l*(*i*,*j*) is the shortest path between node i and j through the network [24].

The mean distance to the root node is the mean of the shortest paths from node i to the root node.

$$\langle D_{root}\rangle =\frac{1}{N}\sum_{i=1}^{N}l(i,1)$$

The mean distance between every node is calculated in much the same way.

$$\langle D\rangle =\frac{1}{N^{2}}\sum_{i=1}^{N}\sum_{j=1}^{N}l(i,j)$$

For finding polygons in the network, we follow the calculation of Alon [21]. In the following, A is the adjacency matrix, D is the degree matrix for the network, and |E| is the amount of edges in the network. *n*_*G*_(*C*_3_),*n*_*G*_(*C*_4_), and *n*_*G*_(*C*_5_) are the number of triangles, squares, and pentagons in the network, respectively. The following calculations assume an undirected network.

$$n_{G}(C_{3})=\frac{1}{6}tr(A^{3})$$

$$n_{G}(C_{4})=\frac{1}{8}\left(tr\left(A^{4}\right)-4\sum_{i=1}^{N}(\begin{array}{c} D(i,i)\\ 2 \end{array})-2\left|E\right|\right)$$

$$n_{G}(C_{5})=\frac{1}{10}\left(tr\left(A^{5}\right)-5\sum_{i=1}^{N}\left[\left(A^{3}(i,i))(D(i,i)-2\right)]-30n_{G}(C_{3}\right)\right)$$

Here, *D*(*i*,*i*) is the degree of node i, and tr (…) is the trace of the matrix. The code calculating all the above network properties has been written in MATLAB 2014a and is in the supporting information as “NetworkProp.m”.

### Adaptation of duct diameter to flux (S11 Fig)

The expected adaptation of duct diameter in response to flux of pancreatic fluid is inspired by previous work in blood vessels [31], satisfying the following equation:

$$\frac{\partial d(t)}{\partial t}=K[\tau (t)-\tau_{desired}]d(t),$$

where d is the duct diameter at time t, K is an adaption constant, and τ is the shear stress experienced by the duct as a result of the fluid flow. In our setup, we consider the system only at steady state, and so

$$\frac{\partial d(t_{ss})}{\partial t}=0\overset{yields}{\to }\tau (t_{ss})=\tau_{desired},$$

which means that at steady state, every duct has adapted to have the desired shear stress, whichever that value might be. Shear stress relates to flux and duct diameter though Poiseuille’s equation (assuming a likely laminar flow and a noncompressible fluid)

$$d^{3}=\frac{32\eta Q}{\pi \tau },$$

where η represents fluid viscosity and Q the flux of fluid running through the duct. Assuming that every part of the duct adapts to have the same shear stress (and by that extension pressure), as they are made of the same material, this shows that every duct at steady state will have a diameter that is proportional to the cubic root of the flux.

$$d(t_{ss})\propto \sqrt[{3}]{Q(t_{ss})}$$

It should be noted that Hacking and colleagues [31] show that given two ducts connected in parallel with a constant flow source, one duct is bound to disappear, while the other will attain the ideal diameter for the flow. This theoretical result explains that redundant ducts can be eliminated in the pancreas, as it will have a constant flow source at steady state.

Experimentally, the duct diameter was measured using Imaris at 27 random nonredundant ducts distributed over the network of an E18.5 pancreas (S11A Fig). The code has been written in MATLAB 2017 and is in the supporting information as “DuctDiameter.m”, “DuctThickness.m”, and “DuctThicknessAnalysis,m”. The analysis shows that the cubic root is a reasonable approximation to the relation between flux, represented as nodes upstream and duct length upstream, and the corresponding duct thickness (S11B and S11C Fig).

### Network diffusion

The network diffusion model works in the following steps:

1. Initiate the system with a network given by its adjacency matrix and coordinates.
2. Define the laplacian for the network as described above.
3. Solve the diffusion scheme as described below, adding fluid at every terminal end of the network and draining fluid at the root node ***ϕ***_*term*_(*t*) = 1,*ϕ*_*root*_(*t*) = 0. Reiterate the diffusion scheme until an approximate steady state has been reached $\sum_{i=1}^{N}\phi (t_{ss}+\Delta t)-\sum_{i=1}^{N}\phi (t_{ss})\approx 0$.
4. The flux from node i to node j at steady state *Q*(*i*,*j*) can now be obtained.

The code applying diffusion to the network has been written in MATLAB 2014a and is in the supporting information as “DiffusionOnNetwork.m”.

Given concentration *ϕ* at node i, which is allowed to freely diffuse, *ϕ* obeys the heat equation

$$\frac{\partial \phi (i,t)}{\partial t}=C\nabla^{2}\phi (i,t),$$

where ∇^2^ is the laplacian, and C is the diffusion coefficient.

On a network, both *ϕ* and ∇^2^ are discretized, and diffusion is constrained to the network links. The heat equation can then be rewritten

$$\frac{d\phi (i)}{dt}=-C\sum_{j}L(i,j)\phi (j),$$

where *L*(*i*,*j*) = ***L*** is the network Laplacian, and *ϕ*(*i*) is the concentration at node i.

The network laplacian for node i can be defined by the node degree d and the neighbors of node i.

$$L(i,j)=d(j)\delta_{ij}-A(i,j)=D(i,j)-A(i,j)=D-A$$

An example of a network diffusion matrix is presented in S9B Fig. In order to solve diffusion numerically, the forward Euler scheme of network diffusion can be used, giving the diffusion scheme

ϕ(t+Δt)=ϕ(t)−CLϕ(t)Δt.

### Network creation model

The network creation model works in the following steps:

1. Choose a random point within radius *r*_*max*_ = max(*d*_*com*_) + 0.5 from the center of mass, where *d*_*com*_ is a vector containing the distances between all nodes and the “center of mass” of the network $d_{com}(i)=\sqrt{{\left(x_{i}-\langle x\rangle \right)}^{2}+{\left(y_{i}-\langle y\rangle \right)}^{2}+{\left(z_{i}-\langle z\rangle \right)}^{2}}$. The chosen point is accepted as a node with probability $P_{accept}=\prod_{i=1}^{N}\left(1-\exp\left(d_{i}\right)\right)$, where *d*_*i*_ is the distance from the random point to node i. Reiterate this step until a node is created.
2. If created, form M + 1 links to other nodes, where M is drawn from a Poisson distribution with mean *λ*. Create these links between the created node to nodes selected at random from a pool of the M + 1 + Δ nearest to the created node.
3. Repeat steps 1 and 2 until the network has the desired size.

The code constructing the random network has been written in MATLAB 2014a and is in the supporting information as “NetworkCreation.m”, as well as the code to initiate the system from an L-system setup instead of two nodes, “RandLsystem.m”.

### Network pruning

The network pruning model works in the following steps:

1. Initiate the system with a network given by its adjacency matrix and flux matrix *Q*.
2. Sort the links according to their flux. Remove the link from the network if its removal does not fragment the network.
3. Rerun “DiffusionOnNetwork”. Repeat steps 1 and 2 until no link can be removed without fragmenting the network.

The code applying network pruning to the network has been written in MATLAB 2014a and is in the supporting information as “PruneBasedOnFlux.m”.

A file named “ExampleScript.m” demonstrates how all the above scripts work in concert.

## Supporting information

S1 Fig Method for digitizing the pancreas network. (A) The network is digitized by mapping the terminal end and intersections of the network and connecting them through the ductal structure. All the digitized networks are based on 3D segmentation, and the resulting networks are also 3D in the spatial sense. (B) An example of the technique applied on a sample network is shown in 2D for clarity.
(TIF)

S2 Fig Determining the dimension of the pancreas networks. Step-by-step instructions on how to obtain the dimension of a given network. (A) The cumulative histogram of the 10th node of the ventral pancreas E14.5 1 network. The marked area indicates the data used for further analysis of network dimension. (B) The selected data in a log-log plot. The slope of the fitted curve is the dimension of the network from the perspective of the given node. The code file “DimFit” is provided in S1 Data. E, embryonic day.
(TIF)

S3 Fig Mapping the whole ventral pancreas E18.5 causes a slight shift in network properties. (A) Part of the E18.5 1 pancreas with network properties that appear in Fig 2. (B) The fully mapped E18.5 1 ventral pancreas. The network only experiences a slight shift in most properties when fully mapped, with the exception of network performance. Digitized data and code files “Import_Experimental_data”, “ConvertToAdjMat”, “ConvertToAdjList”, “NetworkProp”, “NetworkShapes”, “FindTriangles”, “PlotNetwork”,“Remove_kinks” are provided in S1 Data. E, embryonic day.
(TIF)

S4 Fig Location of triangles in the E14.5 networks. The triangles are highlighted in red, while the root node is highlighted in green in the networks for (A) E14.5 1, (B) E14.5 2, and (C) E14.5 3. (D) Histogram showing the triangles’ distance to the root node for the three networks. Digitized data and code files “Import_Experimental_data”, “ConvertToAdjMat”, “ConvertToAdjList”, “FindTriangles”, and “PlotNetwork”,”Remove_kinks” are provided in S1 Data. E, embryonic day.
(TIF)

S5 Fig Random networks based on the in vivo and in vitro networks. < …> denotes the average of a value for every node in the network. C is the clustering coefficient for a given node. k is node degree. D_root_ is a node's distance to the root node through the network. L_tot_ is the total amount of link length in the entire network. Errors represent SEM, with *n* = 100. Digitized data and code files “Import_Experimental_data”, “FindTriangles”, “NetworkProp”, “NetworkShapes”, and “sym_generate_srand_conncomp_Mod” are provided in S1 Data.
(TIF)

S6 Fig Parameter space for the network creation model. < …> denotes the average value for every node in the network. k is node degree. Errors represent SEM, with *n* = 100.
(TIF)

S7 Fig Parameter space of the network creation model. (A) *p*-values from *t* test comparisons between the created in silico network and the in vivo E12.5 networks. Dark red indicates the parameter space in which the in silico model is significantly different from the ventral pancreas E12.5 network. (B) The above pass/fail data combined into one plot. Pass/fail is determined by *t* test with *p* = 0.05 significance and Bonferroni corrections for multiple hypothesis testing. E, embryonic day
(TIF)

S8 Fig Effect of an initial L-system on in silico generated networks. Distribution of polygonal features for networks generated as described in the supporting information but with a varying number of initial nodes connected in a spatially random L-system. The “fully random” results correspond to the “in silico” results in Fig 3C. The generated networks all consist of approximately 320 nodes. Error bars represent SEM, *n* = 10,000. The code files “RandLSystem”, “ConvertToAdjMat”, “ConvertToAdjList”, “NetworkProp”, “NetworkShapes”, “FindTriangles”, “Remove_kinks” are provided in S1 Data.
(TIF)

S9 Fig Description of diffusion-based pruning. (A) Step-by-step instructions on how to prune a given network for both the flux-based and random pruning. (B) Example of diffusion matrix and associated network.
(TIF)

S10 Fig Flux-based pruning of the ventral pancreas E14.5 1 and E14.5 3. (A) The logarithm of the normalized flux at steady state of the pancreas networks. Thicker links indicate a higher flux. The highest flux is closest to the exit, with some interlinking nodes having very low flux. The links highlighted red are pruned by the pruning mechanism of least flux. (B) The pruning events’ distance from the exit as pruning progresses for flux-based pruning and random pruning. Digitized data and code files “Import_Experimental_data”, “DiffusionOnNetwork”, “PruneBasedOnFlux”, “SnapShot”, “ConvertToAdjMat”, “ConvertToAdjList”, “NetworkProp”, “NetworkShapes”, “FindTriangles”, “Remove_kinks” are provided in S1 Data. E, embryonic day.
(TIF)

S11 Fig Measurements of duct thickness on in vivo E18.5 pancreas. Duct diameter of selected ducts of an E18.5 network. (A) The network visualized along with the measured ducts highlighted in green. (B) Duct diameter as a function of nodes upstream of the given duct. (C) Duct diameter as a function of total duct length upstream. Digitized data and code files “Import_Experimental_data”, “PlotNetwork”, “DuctDiameter”, and “DuctThicknessAnalysis” are provided in S1 Data. E, embryonic day.
(TIF)

S12 Fig Expression of transcripts encoding proteins involved in pancreatic secretion. The families of proteins are color-encoded on the right side. The expression is reported in the pancreas at E10.5, E12.5, and E14.5. The rows highlighted in gray correspond to family members with reported function in adult pancreas secretion.
(TIF)

S1 Data Supporting information. **Data and code used to make plots in all figures.** Data are provided as two excel files for every sample: one reporting node ID and position in space and one reporting node connectivity and distances. These excel files were templates used in the code files to generate figures, as indicated in figure legends.
(7Z) Click here for additional data file.

## Abbreviations

3D: three-dimensional
Ano1/Tmem16a: anoctamin-1/transmembrane member 16a
BABB: solution of 1:2 Benzyle Alcohol and Benzyle Benzoate
BICC1: bicaudal C homolog 1
CFTR: cystic fibrosis transmembrane conductance regulator
E: embryonic day
FGF: fibroblast growth factor
HNF1B: hepatocyte nuclear factor 1 beta
HNF-6: hepatocyte nuclear factor 6
Kif3a: kinesin family member 3a
LN: lumen node
MST: minimum spanning tree
NR5A2: nuclear receptor subfamily 5 group A member 2
PBS: phosphate-buffered saline
RhoA: Ras homolog gene family member A
Slc26a6: solute carrier family 26 member 6
