# Supplementary figures and images for: Deconstructing the principles of ductal network formation in the pancreas
Source: PLoS Biol. 2018 Jul 26;16(7):e2002842. doi: 10.1371/journal.pbio.2002842 (PMC6080801; doi:10.1371/journal.pbio.2002842)

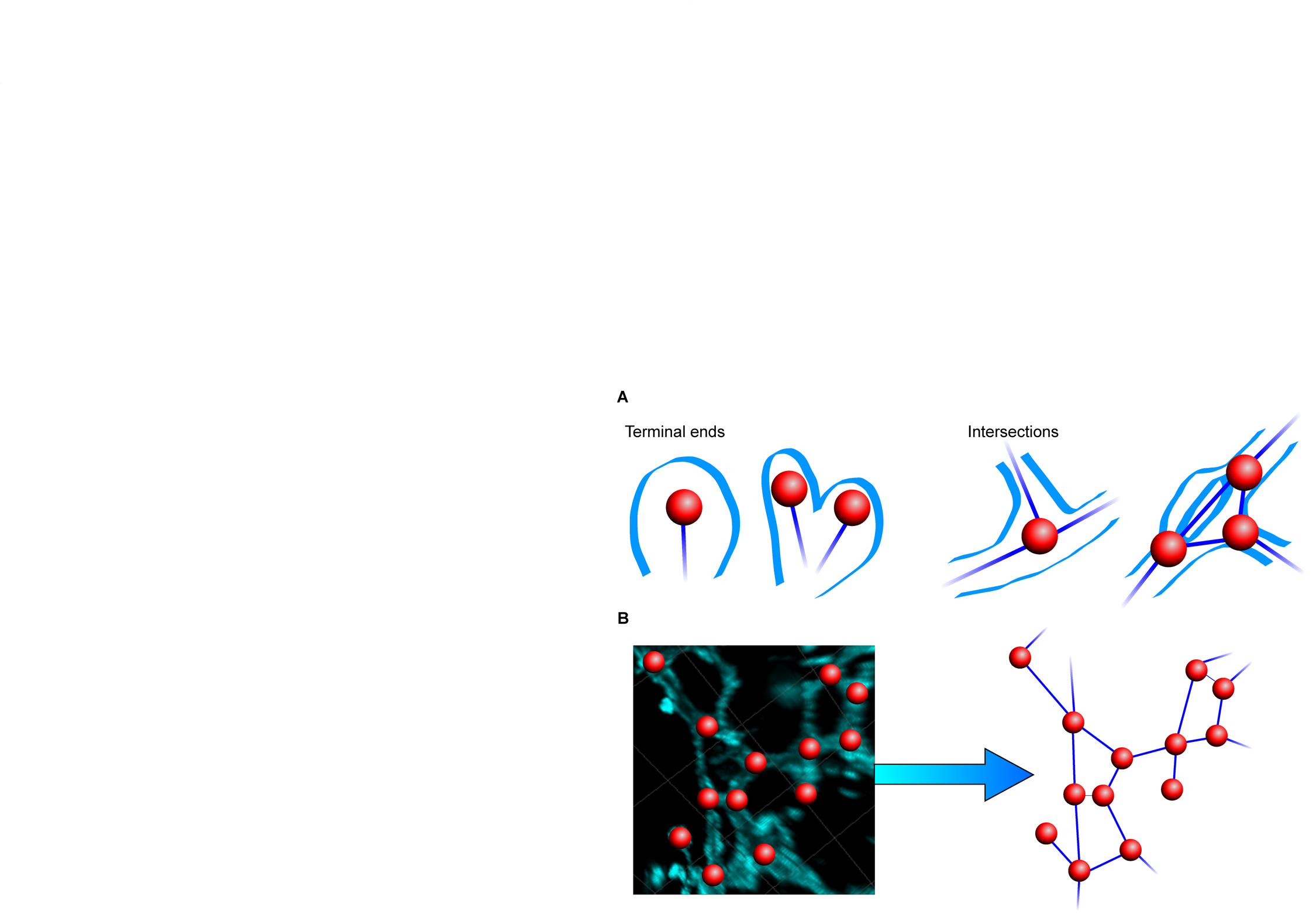

Supplement: S1 Fig — (A) The network is digitized by mapping the terminal end and intersections of the network and connecting them through the ductal structure. All the digitized networks are based on 3D segmentation, and the resulting networks are also 3D in the spatial sense. (B) An example of the technique applied on a sample network is shown in 2D for clarity. (TIF) [file pbio.2002842.s001.tif]

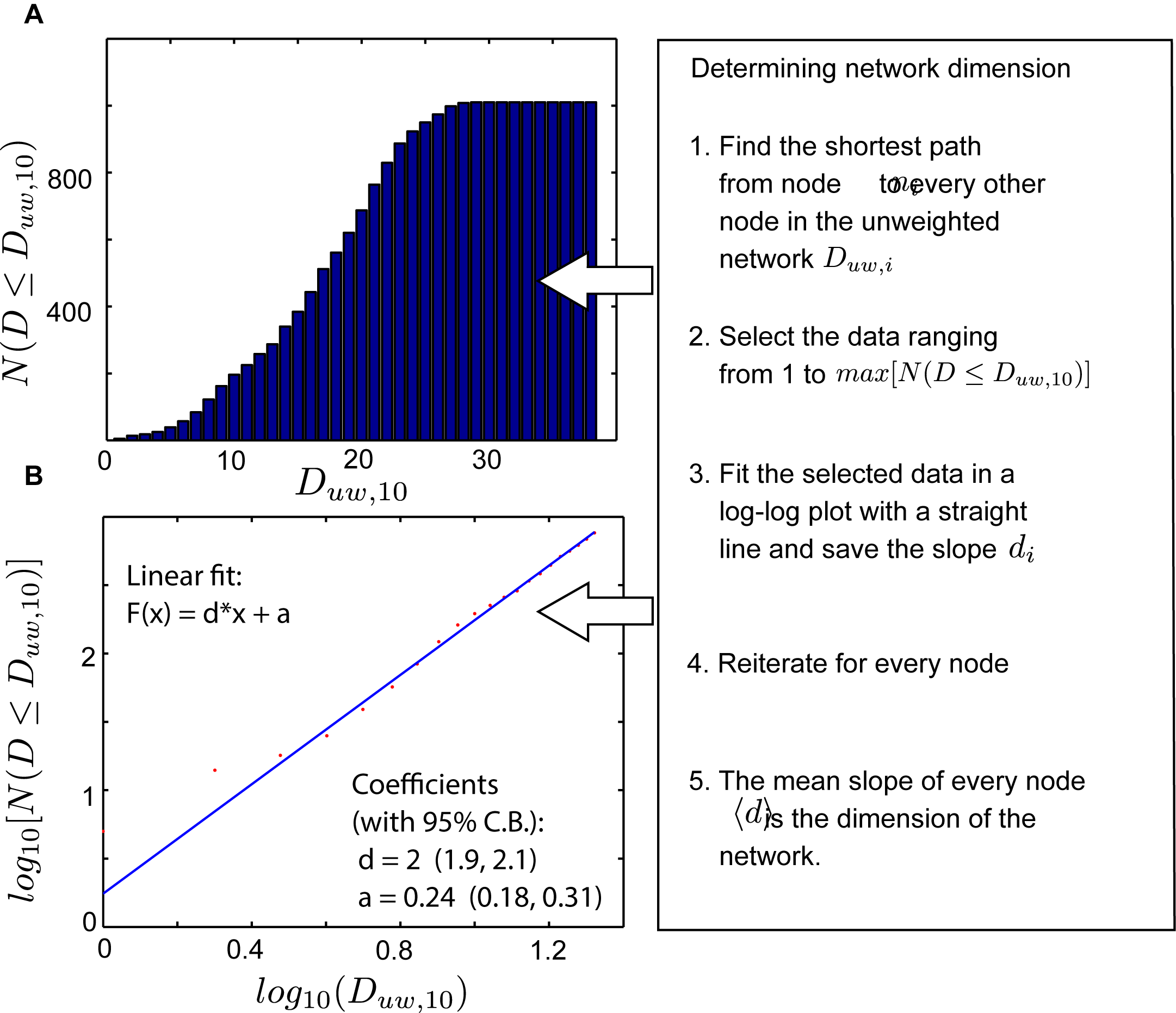

Supplement: S2 Fig — Step-by-step instructions on how to obtain the dimension of a given network. (A) The cumulative histogram of the 10th node of the ventral pancreas E14.5 1 network. The marked area indicates the data used for further analysis of network dimension. (B) The selected data in a log-log plot. The slope of the fitted curve is the dimension of the network from the perspective of the given node. The code file “DimFit” is provided in S1 Data. E, embryonic day. (TIF) [file pbio.2002842.s002.tif]

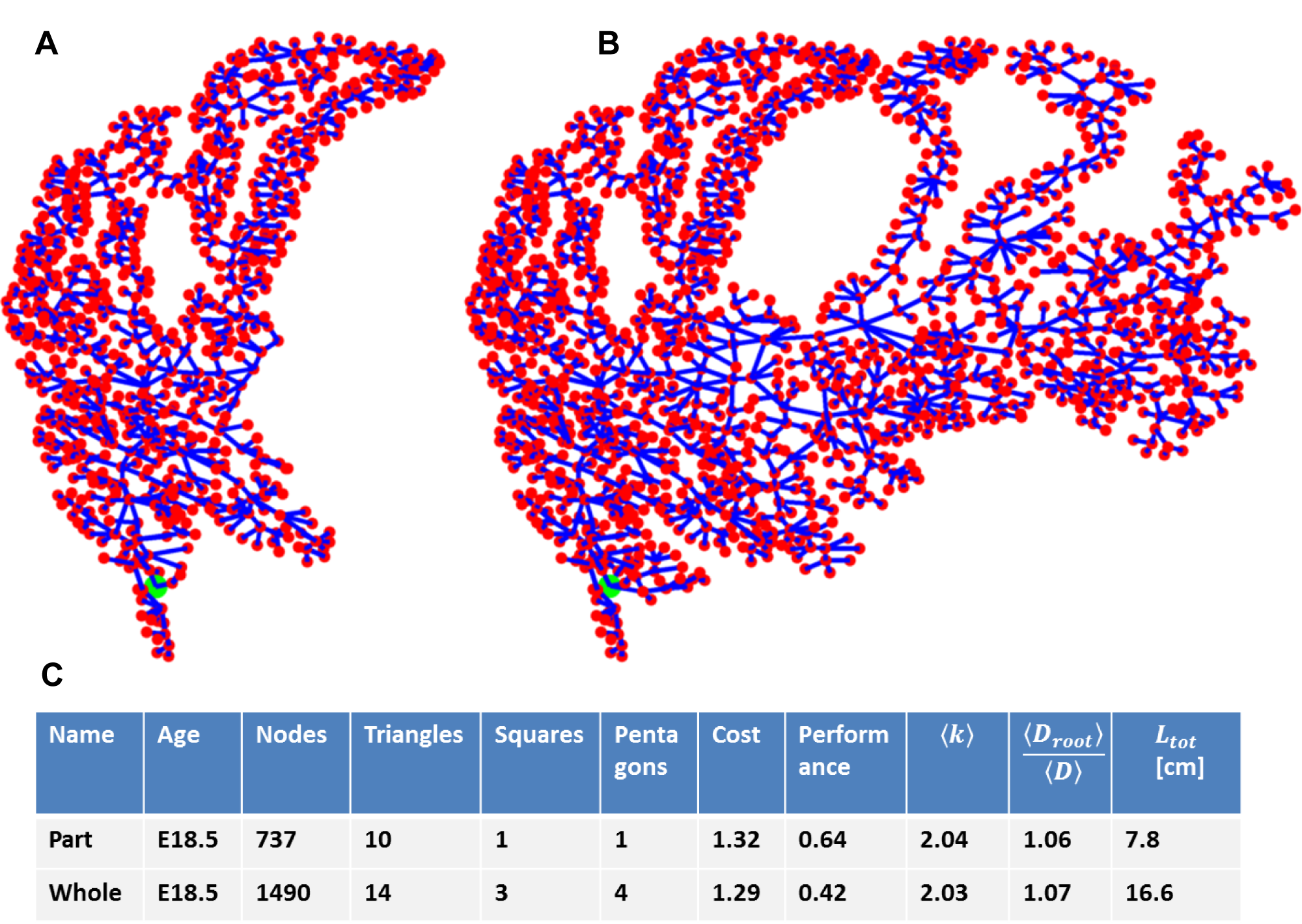

Supplement: S3 Fig — (A) Part of the E18.5 1 pancreas with network properties that appear in Fig 2. (B) The fully mapped E18.5 1 ventral pancreas. The network only experiences a slight shift in most properties when fully mapped, with the exception of network performance. Digitized data and code files “Import_Experimental_data”, “ConvertToAdjMat”, “ConvertToAdjList”, “NetworkProp”, “NetworkShapes”, “FindTriangles”, “PlotNetwork”,“Remove_kinks” are provided in S1 Data. E, embryonic day. (TIF) [file pbio.2002842.s003.tif]

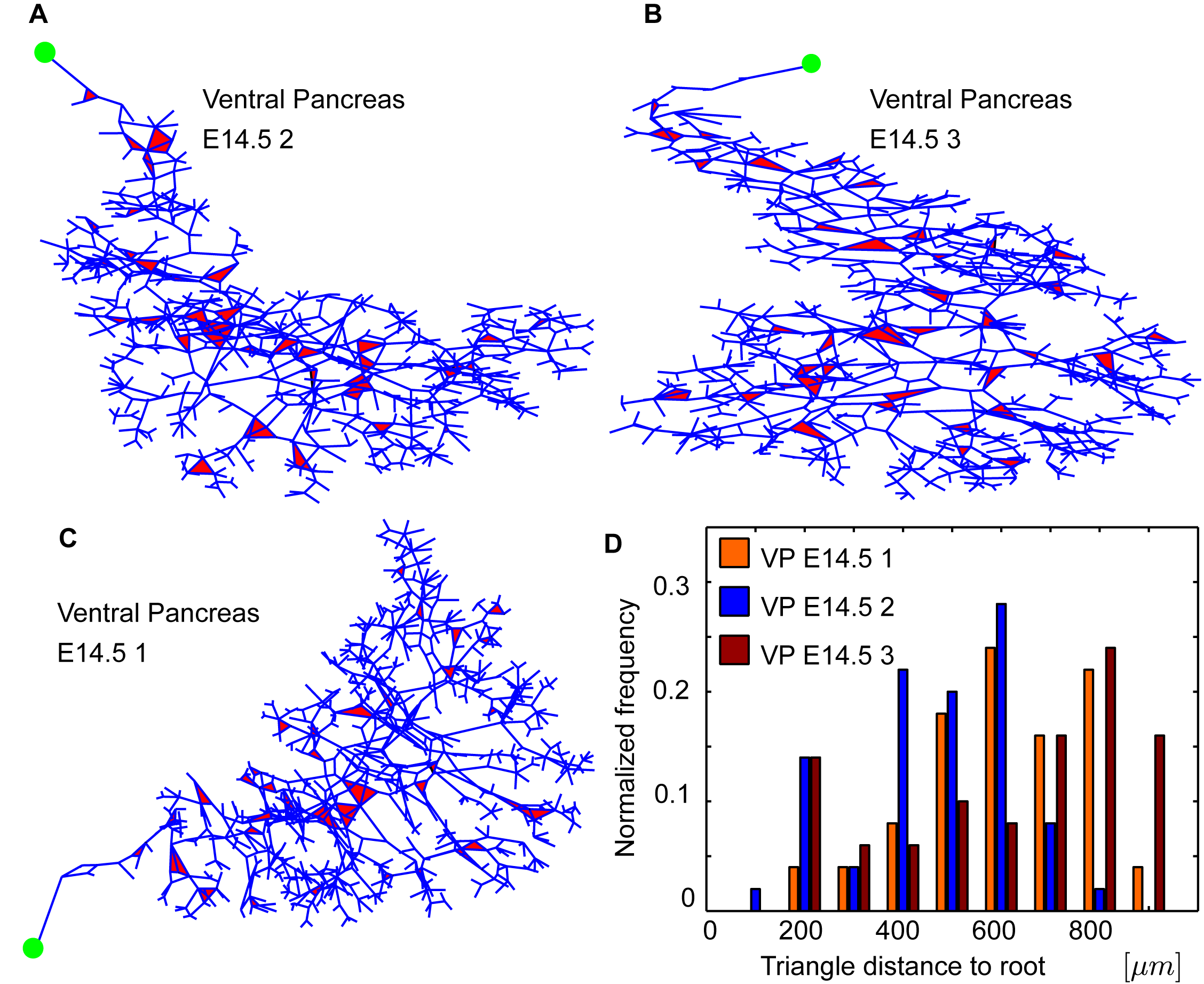

Supplement: S4 Fig — The triangles are highlighted in red, while the root node is highlighted in green in the networks for (A) E14.5 1, (B) E14.5 2, and (C) E14.5 3. (D) Histogram showing the triangles’ distance to the root node for the three networks. Digitized data and code files “Import_Experimental_data”, “ConvertToAdjMat”, “ConvertToAdjList”, “FindTriangles”, and “PlotNetwork”,”Remove_kinks” are provided in S1 Data. E, embryonic day. (TIF) [file pbio.2002842.s004.tif]

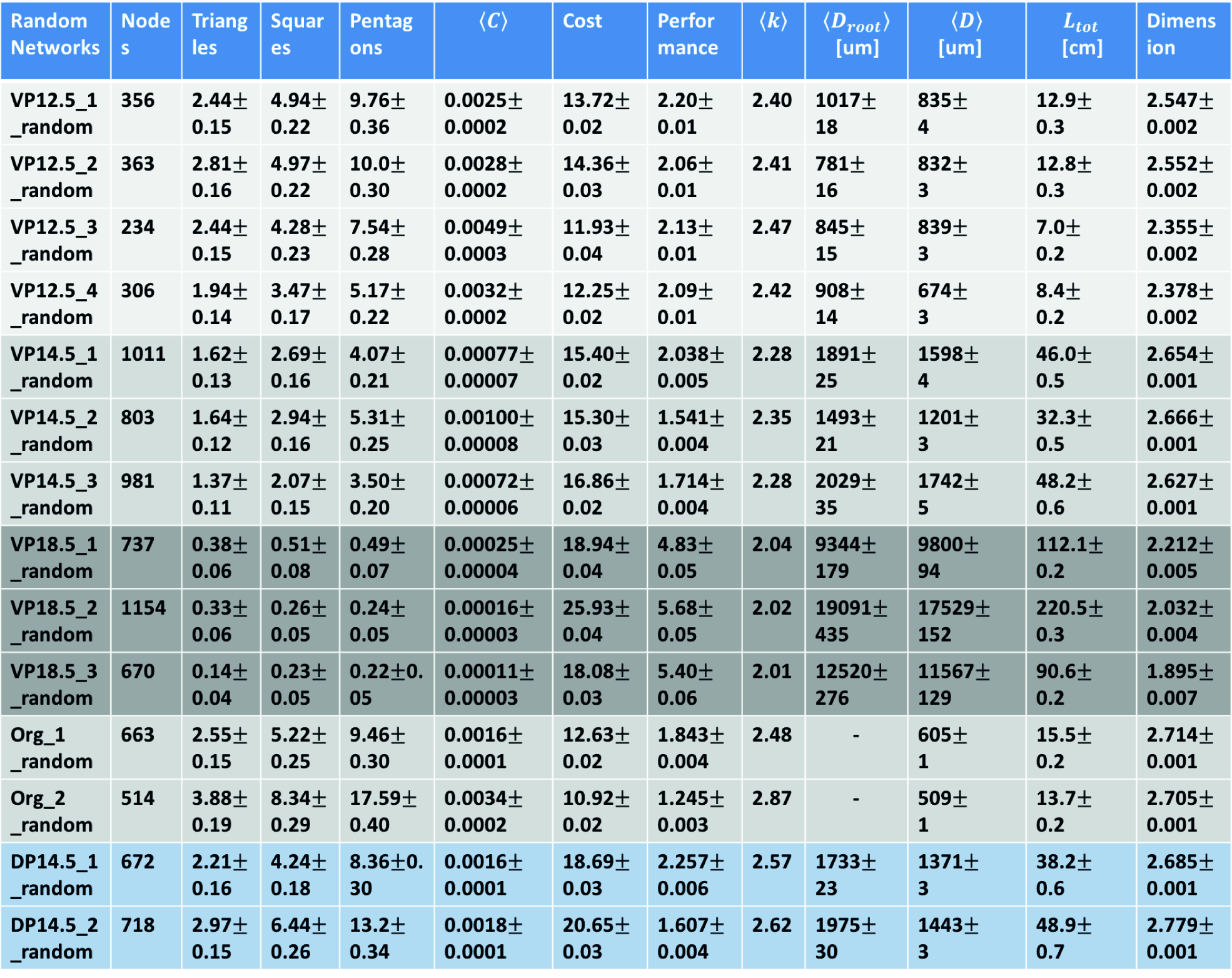

Supplement: S5 Fig — < …> denotes the average of a value for every node in the network. C is the clustering coefficient for a given node. k is node degree. Droot is a node's distance to the root node through the network. Ltot is the total amount of link length in the entire network. Errors represent SEM, with n = 100. Digitized data and code files “Import_Experimental_data”, “FindTriangles”, “NetworkProp”, “NetworkShapes”, and “sym_generate_srand_conncomp_Mod” are provided in S1 Data. (TIF) [file pbio.2002842.s005.tif]

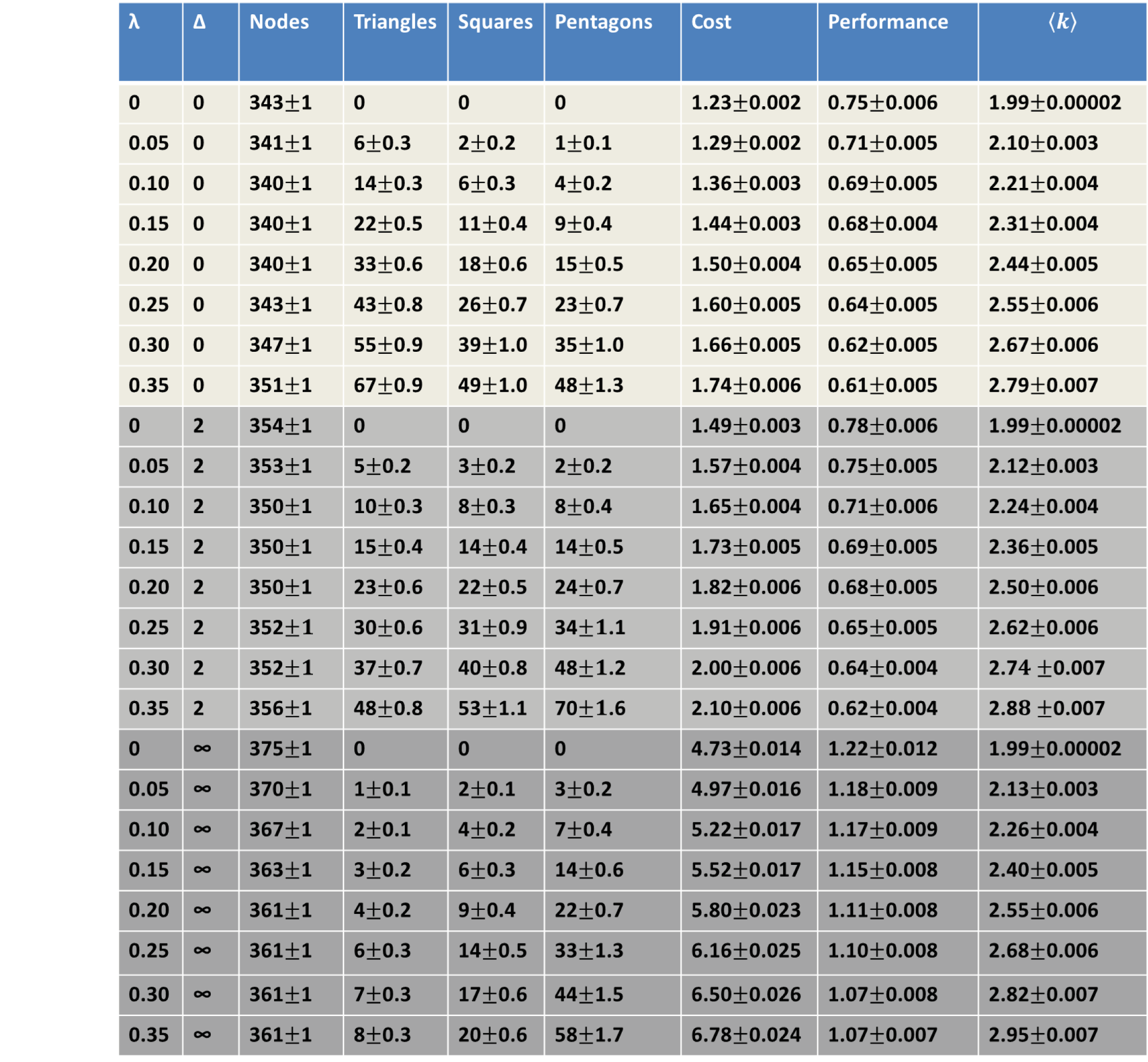

Supplement: S6 Fig — < …> denotes the average value for every node in the network. k is node degree. Errors represent SEM, with n = 100. (TIF) [file pbio.2002842.s006.tif]

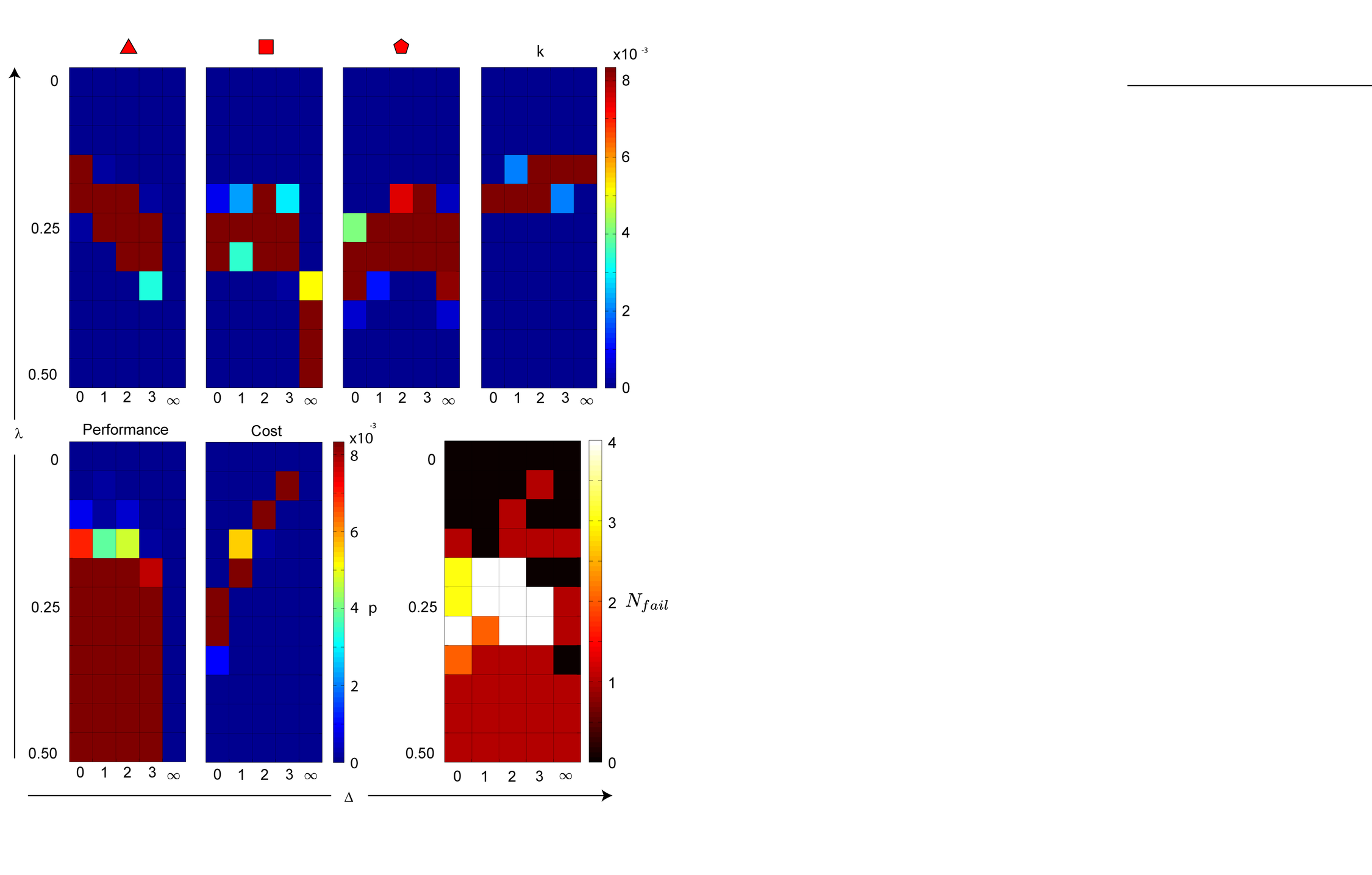

Supplement: S7 Fig — (A) p-values from t test comparisons between the created in silico network and the in vivo E12.5 networks. Dark red indicates the parameter space in which the in silico model is significantly different from the ventral pancreas E12.5 network. (B) The above pass/fail data combined into one plot. Pass/fail is determined by t test with p = 0.05 significance and Bonferroni corrections for multiple hypothesis testing. E, embryonic day (TIF) [file pbio.2002842.s007.tif]

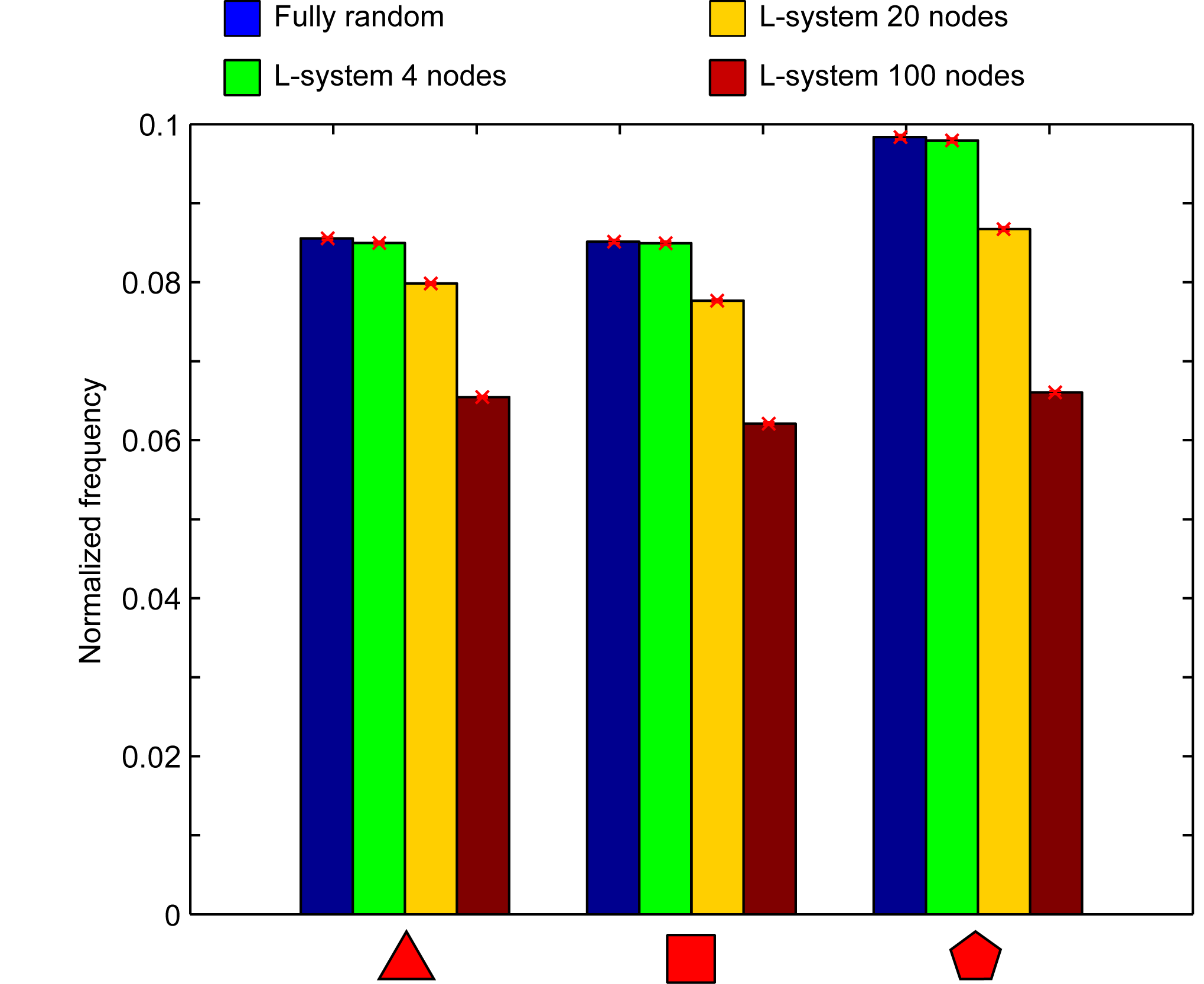

Supplement: S8 Fig — Distribution of polygonal features for networks generated as described in the supporting information but with a varying number of initial nodes connected in a spatially random L-system. The “fully random” results correspond to the “in silico” results in Fig 3C. The generated networks all consist of approximately 320 nodes. Error bars represent SEM, n = 10,000. The code files “RandLSystem”, “ConvertToAdjMat”, “ConvertToAdjList”, “NetworkProp”, “NetworkShapes”, “FindTriangles”, “Remove_kinks” are provided in S1 Data. (TIF) [file pbio.2002842.s008.tif]

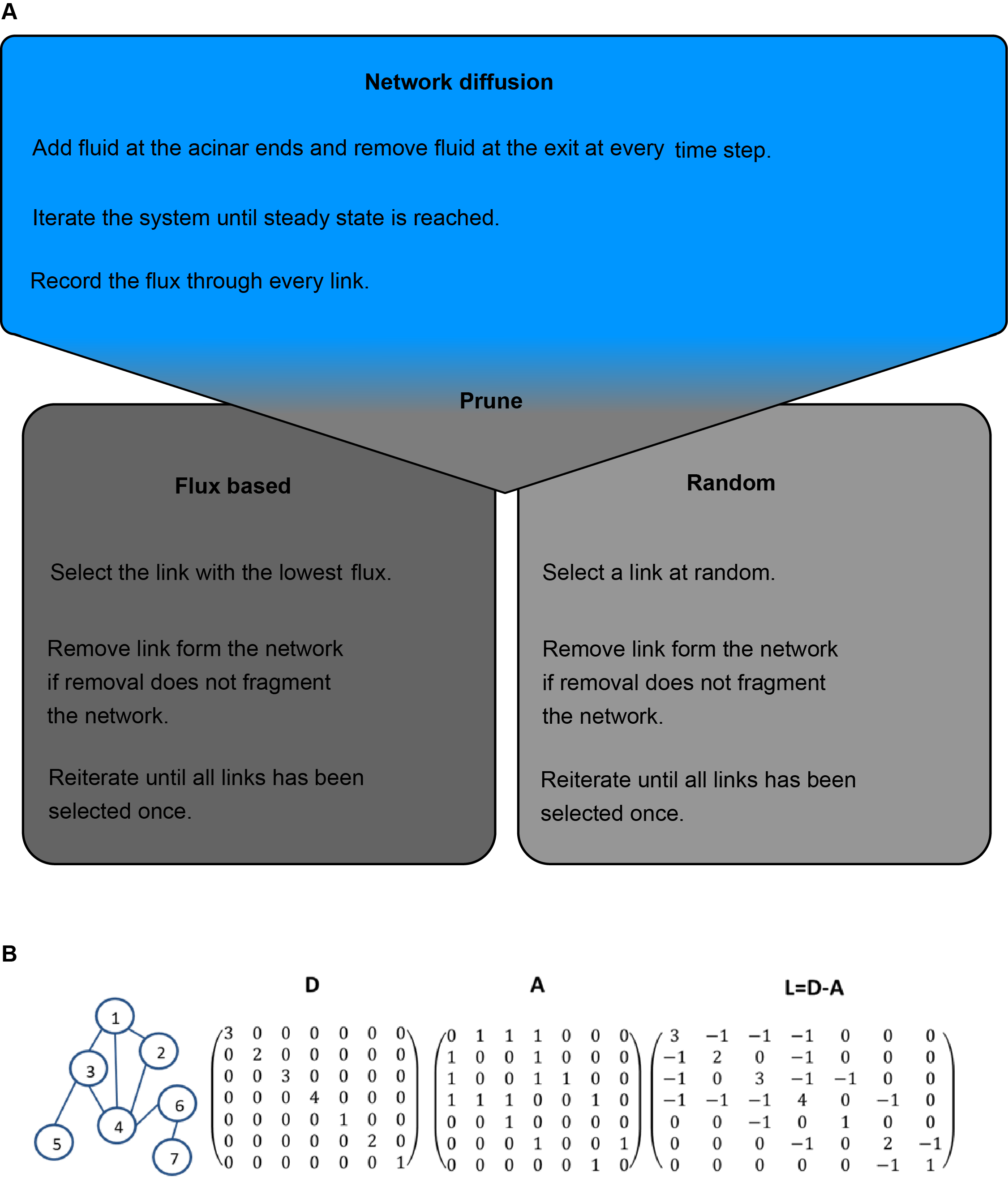

Supplement: S9 Fig — (A) Step-by-step instructions on how to prune a given network for both the flux-based and random pruning. (B) Example of diffusion matrix and associated network. (TIF) [file pbio.2002842.s009.tif]

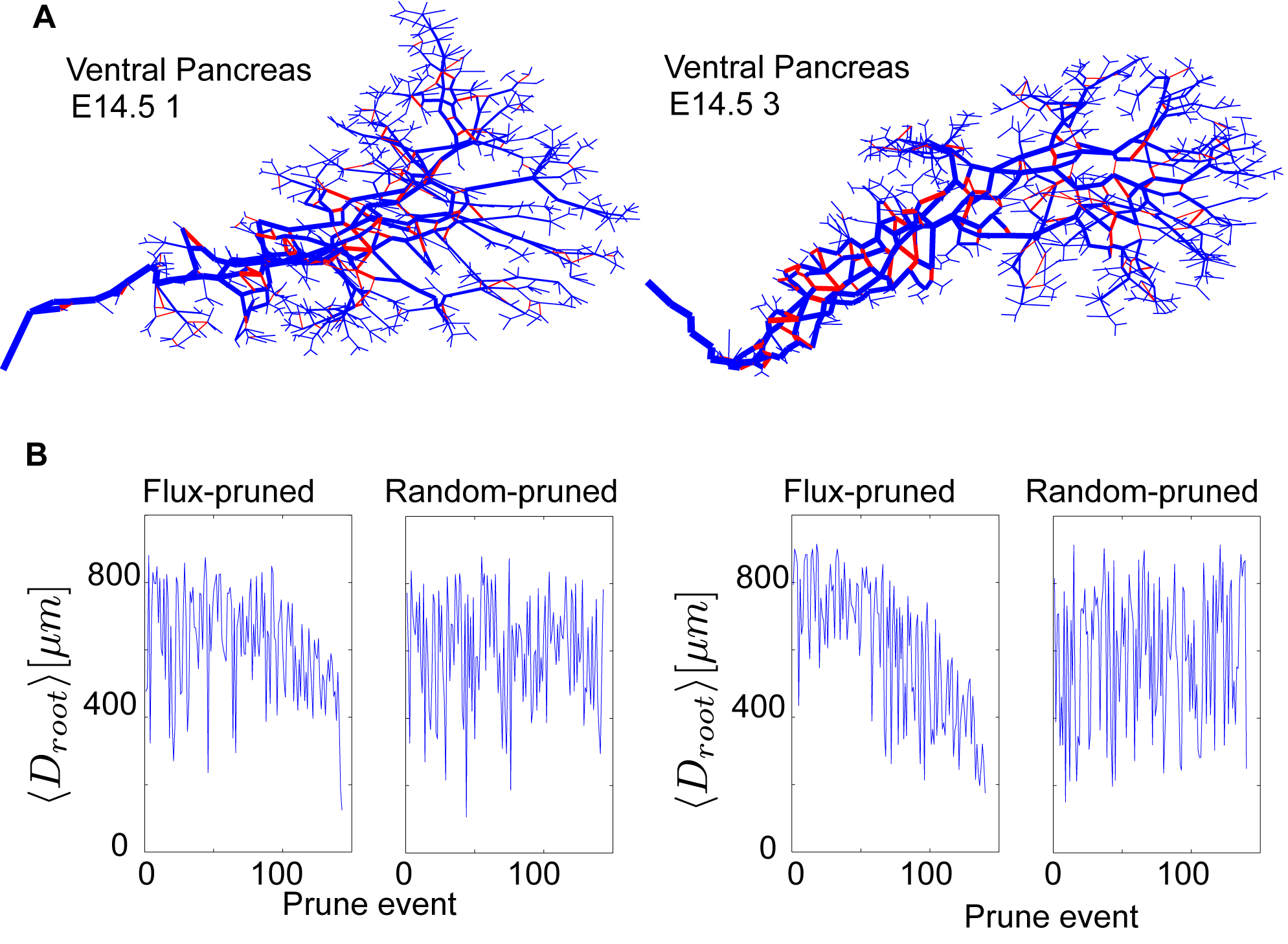

Supplement: S10 Fig — (A) The logarithm of the normalized flux at steady state of the pancreas networks. Thicker links indicate a higher flux. The highest flux is closest to the exit, with some interlinking nodes having very low flux. The links highlighted red are pruned by the pruning mechanism of least flux. (B) The pruning events’ distance from the exit as pruning progresses for flux-based pruning and random pruning. Digitized data and code files “Import_Experimental_data”, “DiffusionOnNetwork”, “PruneBasedOnFlux”, “SnapShot”, “ConvertToAdjMat”, “ConvertToAdjList”, “NetworkProp”, “NetworkShapes”, “FindTriangles”, “Remove_kinks” are provided in S1 Data. E, embryonic day. (TIF) [file pbio.2002842.s010.tif]

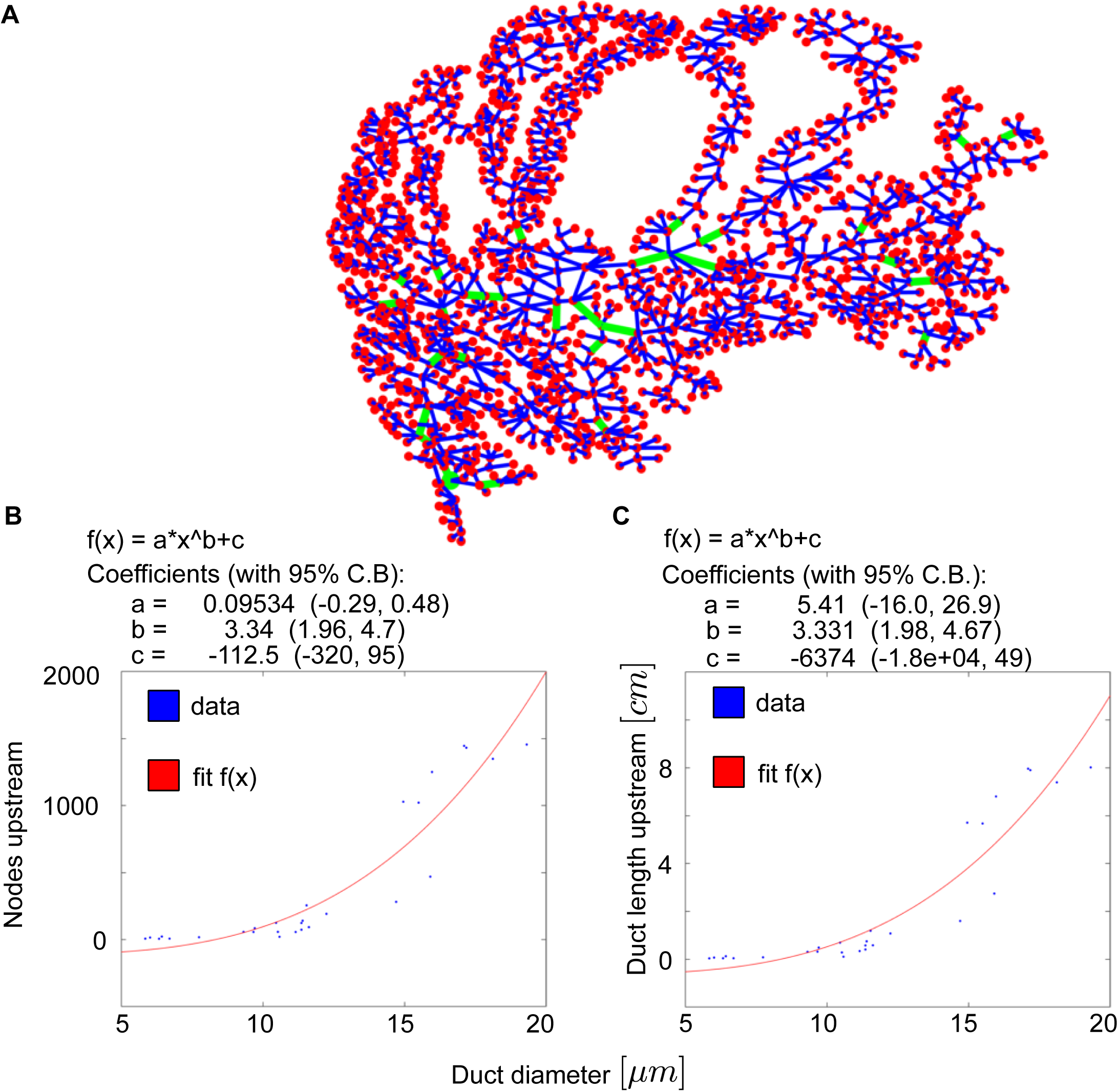

Supplement: S11 Fig — Duct diameter of selected ducts of an E18.5 network. (A) The network visualized along with the measured ducts highlighted in green. (B) Duct diameter as a function of nodes upstream of the given duct. (C) Duct diameter as a function of total duct length upstream. Digitized data and code files “Import_Experimental_data”, “PlotNetwork”, “DuctDiameter”, and “DuctThicknessAnalysis” are provided in S1 Data. E, embryonic day. (TIF) [file pbio.2002842.s011.tif]

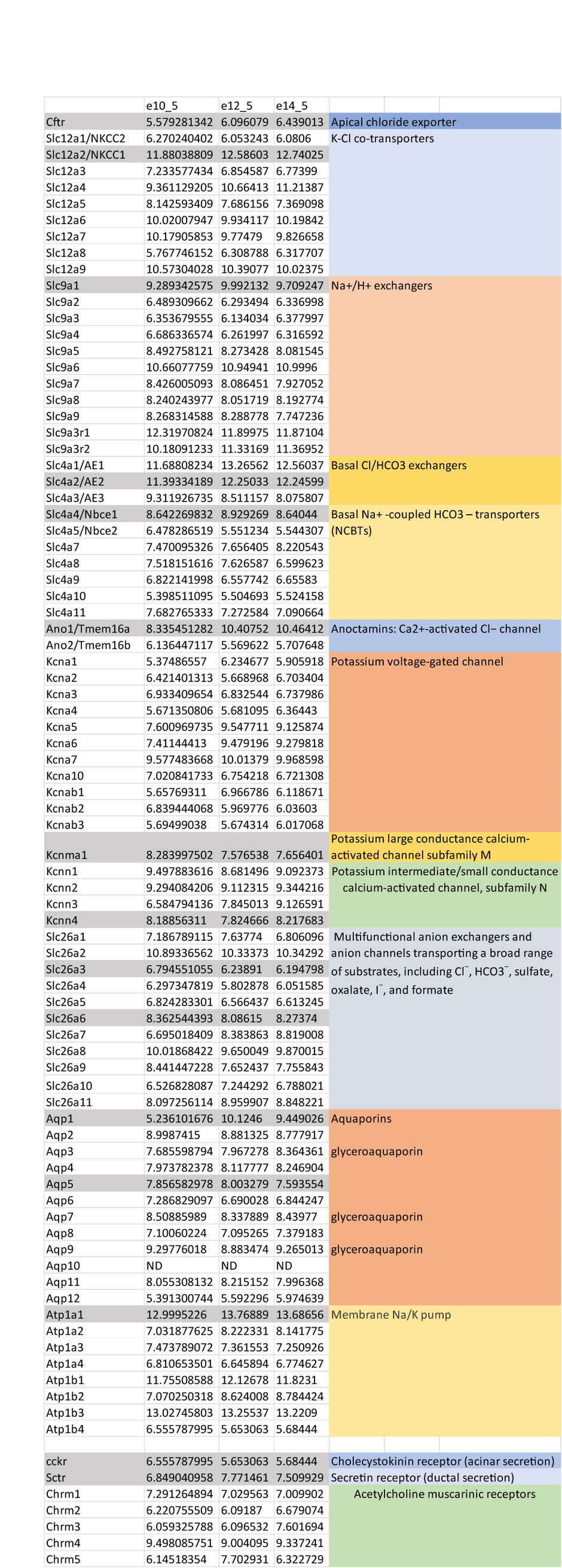

Supplement: S12 Fig — The families of proteins are color-encoded on the right side. The expression is reported in the pancreas at E10.5, E12.5, and E14.5. The rows highlighted in gray correspond to family members with reported function in adult pancreas secretion. (TIF) [file pbio.2002842.s012.tif]
